# Wilson’s disease-associated gut dysbiosis: novel insights into microbial functional alterations, virulence changes, and resistance markers

**DOI:** 10.3389/fmicb.2025.1714276

**Published:** 2026-01-15

**Authors:** Taohua Wei, Nannan Qian, Han Wang, Yuqi Song, Weiqi Wang, Yangyang Li, Zihao Zhao, Fulin Xu, Wenming Yang

**Affiliations:** 1 The First Affiliated Hospital of Anhui University of Chinese Medicine, Hefei, Anhui, China; 2 Center for Xin’an Medicine and Modernization of Traditional Chinese Medicine, Institute of Health and Medicine, Hefei Comprehensive National Science Center, Hefei, Anhui, China; 3 Key Laboratory of Xin’An Medicine, Ministry of Education, Hefei, Anhui, China

**Keywords:** Wilson’s disease, gut metagenome, mobile genetic elements (MGEs), antibiotic resistance genes (ARGs), virulence factors (VFs)

## Abstract

**Background:**

Although the gut microbiota is associated with a variety of metabolic, inflammatory, and neurological disorders through microbial dysbiosis, current studies on the gut microbiota in Wilson’s disease (WD) remain limited. Critical gaps exist in understanding the roles of key functional microbial factors in WD pathogenesis, which hinders the acquisition of mechanistic insights into this disease.

**Objective:**

This study aims to characterize alterations in the gut microbiome associated with WD, with a particular emphasis on virulence factors (VFs) and antibiotic resistance genes (ARGs), as well as functional mobile genetic elements (MGEs), in order to elucidate their potential roles in disease progression and clinical manifestations.

**Methods:**

We analyzed fecal samples from 37 patients with WD and 33 healthy controls (HCs) using metagenomic sequencing, with a specific focus on examining virulence gene profiles and antibiotic resistance patterns and MGE composition in relation to liver function markers.

**Results:**

Beta diversity analysis revealed significant differences in the gut microbial community structure between patients with WD and HCs, and a distinct set of microbial taxa was identified that showed significant associations with clinical indicators. A gut microbial co-occurrence network identified key species playing central roles in the microbial community structure, including *Prevotella stercorea*, *Firmicutes bacterium* CAG 110, *Bacteroides salyersiae*, *Lactococcus petauri, Streptococcus cristatus, Actinomyces sp*. HMSC035G02, and *Streptococcus viridans.* Widespread functional dysbiosis was detected across multiple biological levels in patients with WD, with significant correlations identified between these microbial alterations and clinical indicators. Significant disruptions were identified in key metabolic pathways, including the Pentose Phosphate Pathway, Pyruvate Metabolism, and Starch and Sucrose Metabolism, which were associated with the dysregulation of carbohydrate-active enzymes (CAZymes). These alterations showed significant correlations with clinical markers of liver dysfunction (e.g., procollagen III N-terminal peptide PIIINP, aspartate transaminase/alanine transaminase AST/ALT). A total of 54 virulence factor (VF) genes exhibited differential abundance in WD, with 36 genes depleted and 18 enriched. Notably, these included colibactin genes (*clbB, clbH*) from *Escherichia coli* and type IV secretion system genes (*aec19, pilB*). These VFs were significantly associated with indicators of liver function (e.g., bilirubin levels) and coagulation abnormalities. Among the detected antibiotic resistance genes (ARGs), 21 exhibited disease-specific patterns in WD, notably *tetQ* (encoding tetracycline resistance), *ErmB* (conferring macrolide resistance), and c*fxA6* (mediating cephamycin resistance). Furthermore, ARG profiles were associated with *Bifidobacterium* enrichment and showed significant correlations with lipid metabolism markers [e.g., triglycerides (TG), high-density lipoprotein cholesterol (HDL-C)]. Critically, we identified significant enrichment of 60 functional mobile genetic elements (MGEs) in WD, spanning categories involved in DNA replication/repair, phage activity, and conjugative transfer, indicating heightened genomic plasticity and horizontal gene transfer potential. Strikingly, correlation network analysis revealed strong and specific co-occurrence between key ARGs (e.g., ErmX) and defined suites of MGEs, suggesting MGE-facilitated dissemination of resistance determinants.

**Conclusion:**

Wilson’s disease (WD) patients exhibit significant alterations in gut microbial community structure and functional dysbiosis, wherein the enrichment of virulence genes (such as colibactin genes *clbB*/*clbH*) and the specific antibiotic resistance genes (such as *tetQ* and *ErmB*), and the activation of mobile genetic elements are closely associated with clinical indicators including liver function impairment, coagulation abnormalities, and lipid metabolism disorders.

## Introduction

Wilson’s disease (WD), also referred to as hepatolenticular degeneration, is a rare autosomal recessive disorder characterized by defective copper metabolism, which results from pathogenic mutations in the *ATP7B* gene encoding a copper-transporting P-type ATPase. It has an estimated global prevalence of 1 in 30,000 to 50,000 individuals (Sandahl et al., 2019). The accumulation of copper ions in multiple organs leads to tissue damage and pathological alterations in affected systems. Common clinical manifestations include hepatic dysfunction, neurological impairments, psychiatric disturbances, and renal abnormalities (Mohr et al., 2023).

The gut microbiota is integral to human health, contributing to essential physiological processes including dietary metabolism, synthesis of fundamental metabolites (e.g., vitamins), preservation of intestinal barrier integrity, pathogen antagonism, and xenobiotic metabolism. In addition, the gut microbiome modulates host immune responses, neurodevelopment, and neural plasticity through the production of bioactive metabolites that exert systemic effects, such as facilitating microglial maturation and maintaining central nervous system homeostasis. Dysbiosis, an imbalance in the composition and functional profile of microbial communities, compromises host immune defenses and enhances susceptibility to infectious diseases. A substantial body of evidence derived from both human clinical studies and animal models indicates that microbial dysbiosis is associated with a wide range of pathological conditions, including metabolic disorders (e.g., diabetes mellitus), inflammatory diseases (e.g., inflammatory bowel disease), neurological disorders (e.g., Parkinson’s disease), and neurodevelopmental conditions (e.g., autism spectrum disorder) (Loh et al., 2024; Aburto and Cryan, 2024). Although gut microbiota alterations in WD have been reported, existing studies primarily rely on 16S rRNA sequencing (Pu et al., 2025; Tang et al., 2025). Moreover the limited metagenomic studies are constrained by insufficient sample sizes (Cai et al., 2024), and there is a critical deficiency in investigations focusing on carbohydrate-active enzymes (CAZymes), antibiotic resistance genes (ARGs), and virulence factor profiles (VFs), which leaves key mechanistic questions unresolved.

Thousands of CAZymes are encoded by microbial genomes, in contrast to humans, who possess only 17 related genes. This disparity suggests that humans lack the complex enzymatic machinery required to degrade various types of complex carbohydrates (Kaoutari et al., 2013; Drula et al., 2021). Therefore, humans depend on symbiotic co-metabolism with microbial communities to derive energy, particularly from indigestible carbohydrates (Onyango et al., 2021). The intestinal microbiota possesses diverse ARGs, enhancing its capacity to withstand antibiotics introduced in clinical settings. Crucially, the dissemination and persistence of ARGs are often facilitated by MGEs, such as plasmids, transposons, and integrative conjugative elements, which drive horizontal gene transfer (HGT) within microbial communities. Microorganisms employ a broad array of VFs to successfully colonize and reproduce within the host, frequently resulting in life-threatening infections (Leitão, 2020). Clarifying the status of CAZymes, ARGs, MGE, and VFs in patients with WD will enhance our understanding of the cellular and molecular mechanisms underlying bacterial pathogenesis in this population. Furthermore, it will aid in evaluating the efficacy of various empirical antibiotic treatments, thereby contributing to the reduction of disease incidence and mortality rates among affected individuals.

In this study, we conducted the first comprehensive metagenomic analysis of the gut microbiome in patients with WD. The research was carried out by a single team of investigators, enabling full control over the study’s design and implementation, ensuring the application of state-of-the-art methodologies, and maintaining consistency throughout the entire process. Utilizing a large sample size and comprehensive shotgun sequencing, we have generated extensive novel insights, revealing widespread dysbiosis in the microbiome of patients with WD, identifying key microbial species contributing to this dysbiosis, and highlighting candidate microbial genes (particularly CAZymes, ARGs, MGE and VFs) and associated pathways in the gut that may play a role in the pathogenic mechanisms of WD.

## Materials and methods

### Ethical approval and patient recruitment

This study was approved by the Ethics Committee of the First Affiliated Hospital of Anhui University of Chinese Medicine (Approval No. 2021MCZQ07). Patients clinically diagnosed with WD were consecutively recruited from August 2021 to September 2023, and underwent comprehensive evaluation at the Department of Neurology in accordance with the recommendations of the EASL Guidelines (EASL, 2012). The diagnosis was based on the Leipzig scoring system (score ≥4) and included the following criteria: age at symptom onset (defined by initial manifestations of WD), clinical presentation categorized as hepatic, neurological, or “other manifestations,” slit-lamp-confirmed Kayser-Fleischer (K-F) rings, and laboratory parameters (serum ceruloplasmin, 24-h urinary copper, and hepatic copper when available). This study employed a feasibility-based convenience sampling strategy due to Wilson’s Disease (WD) being a rare disease.

### Shotgun metagenome sequencing

Fecal sample collection and processing for shotgun metagenomic sequencing were conducted following established protocols. Participants provided fecal samples using sterile collection containers, with parental assistance in transferring the samples to pre-labeled tubes while wearing gloves to maintain aseptic conditions. Samples were immediately flash-frozen and stored at −80 °C to ensure long-term preservation. For shotgun metagenomic sequencing and taxonomic annotation, DNA was extracted from stool samples using the StoolGen DNA Kit (CWBiotech Co., Beijing, China). Library construction was carried out using the TruSeq DNA Sample Preparation Kit (Illumina), and sequencing was conducted on an Illumina HiSeq 4000 platform.

### Metagenome sequencing analysis

Adapter and low-quality sequences were removed from the raw FASTQ files using FASTP (v0.23.0) to ensure data quality (Chen et al., 2018). The reads were trimmed and aligned to the hg19 genome using STAR v2.9.11b (Dobin et al., 2013). Taxonomic profiling of these samples was performed using MetaPhlAn 3.0 (Truong et al., 2017), yielding relative abundances at the levels of phylum, class, order, family, genus, and species (i.e., the percentage each taxon constitutes relative to all detected species within a sample). Functional profiling of gene families and pathways was performed using HUMAnN3 with the UniRef90 database. Mapping of UniRef90 genes to the Gene Ontology (GO), KEGG Orthology (KO), MetaCyc, and Kyoto Encyclopedia of Genes and Genomes (KEGG) pathways was conducted, with pathway abundance analysis based on KO abundance.

### Network analysis of species co-occur

Correlation networks for the WD and control microbiomes were constructed using WGCNA (Langfelder and Horvath, 2008), with species abundance correlations evaluated through the Spearman rank correlation test, with significant correlations defined as |r| > 0.2 and *p* < 0.05. Network degree was calculated using the igraph package, and significant correlations were visualized using Cytoscape (Shannon et al., 2003). Clustering analysis was conducted using the MCODE v1.3 with its default parameter settings.

### Cazy, CARD, VF and Mobile genetic element (MGE) prediction

To characterize CAZymes, ARGs, and VFs, we conducted functional annotation through Diamond blastx (Buchfink et al., 2015) against the following databases: the CAZy (Drula et al., 2021) for carbohydrate-active enzymes; the VFDB (Liu et al., 2022) for virulence factors; and the Comprehensive Antibiotic Resistance Database (CARD, card.mcmaster.ca) (Alcock et al., 2020) for ARO-annotated antibiotic resistance genes. Reads Per Million (RPM) was calculated with non-host total reads as background. Subsequently, differentially abundant genes were identified by applying DEseq2 to read counts and the Wilcoxon rank-sum test to RPM values.

### Correlational network analysis between antibiotic resistance genes and Mobile genetic elements

To explore potential mechanisms for the dissemination of antibiotic resistance, we constructed a correlation network between significantly differential ARGs and MGEs. Spearman correlation coefficients were calculated based on the RPM values of all ARGs and MGEs across all samples. Statistically significant positive correlations (Spearman’s *ρ* ≥ 0.3, *p* < 0.05) were selected to construct an undirected network. This analysis aimed to identify high-confidence ARG-MGE pairs that may indicate physical linkage or co-mobilization potential within the gut microbial community of WD patients.

### Statistical analyses

All statistical analyses were performed using R 4.1.1. These analyses included the calculation of Shannon *α*-diversity; *β*-diversity assessment via principal coordinate analysis (PCoA) and PERMANOVA (Bray–Curtis dissimilarity, vegan v2.5–7); group comparisons using Wilcoxon rank-sum tests; rarefaction analysis to estimate gene richness; and data visualization using the ComplexHeatmap and ggplot2 packages (Gu and Hübschmann, 2022; Wickham, 2009).

## Results

### Bacteria differing in abundance between WD cases and controls

**Figure 1:**
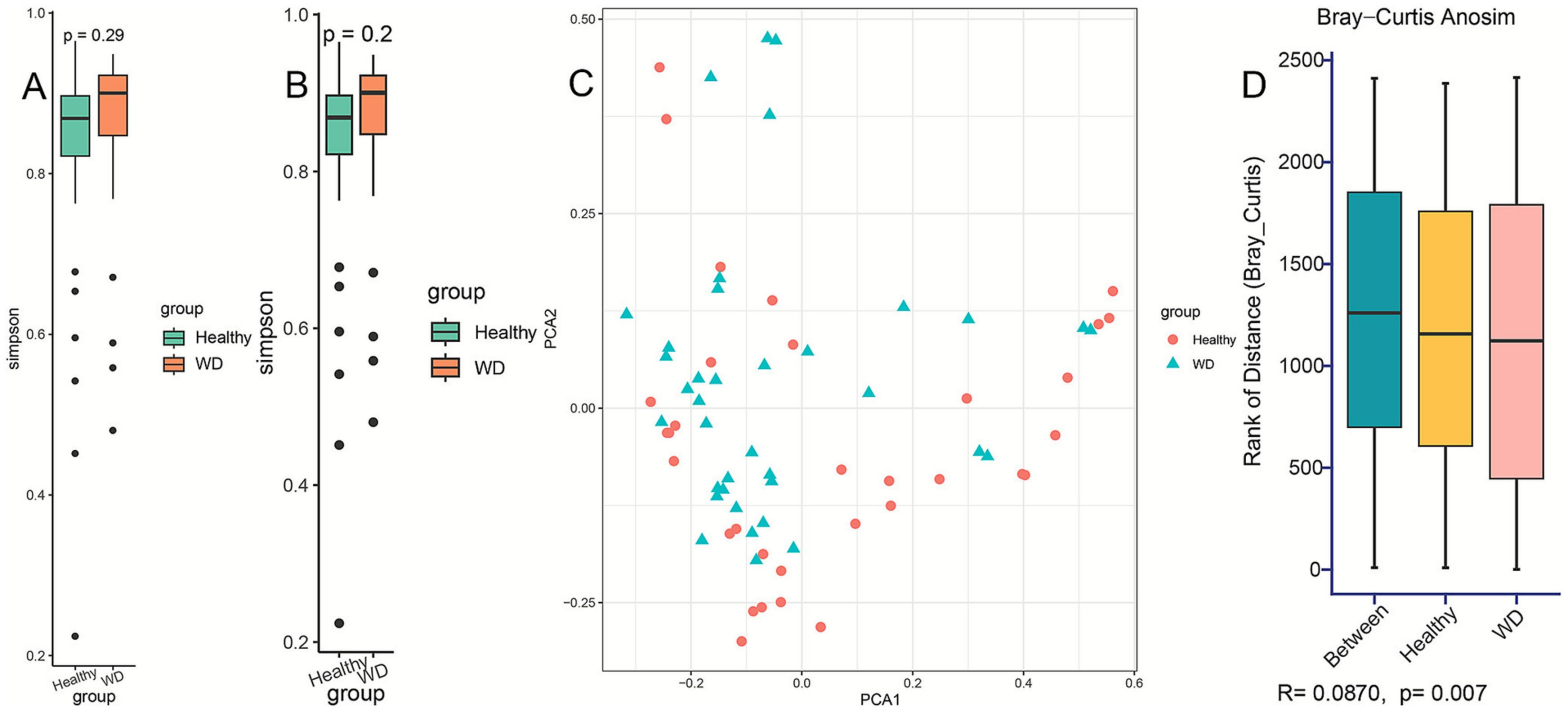
Gut microbiome diversity and composition in Wilson’s disease (WD) versus healthy controls. **(A)** Alpha diversity (Shannon index) showing no significant difference between groups (Wilcoxon test, *p* = 0.29). **(B)** Alpha diversity (Simpson index) showing no significant difference (Wilcoxon test, *p* = 0.2). **(C)** Principal coordinates analysis (PCoA) of Bray-Curtis dissimilarity, revealing significant separation between WD and healthy groups (PERMANOVA, *p* = 0.007). **(D)** ANOSIM analysis confirming distinct community structures (*R* = 0.087, *p* < 0.001). Group labels: Healthy (blue), WD (red). NS, not significant.

To investigate potential dysbiosis of gut bacterial communities in patients with WD, we compared both alpha and beta diversity between the WD and control groups. No significant difference was observed in alpha diversity (Shannon diversity index, *p* = 0.83; Simpson diversity index, *p* = 0.2), which reflects the richness and evenness of the gut microbiome, between the two groups (Figures 1A,B). However, statistically significant differences in beta diversity were observed between the HC and WD groups, as measured by the Bray–Curtis distance metric (*p* = 0.007) (Figures 1C,D) and confirmed by MRPP (*p* = 0.002) and Adonis (*p* = 0.002) tests. Collectively, these results indicate distinct alterations in the gut microbial community structure among patients with WD compared to healthy individuals.

**Figure 2:**
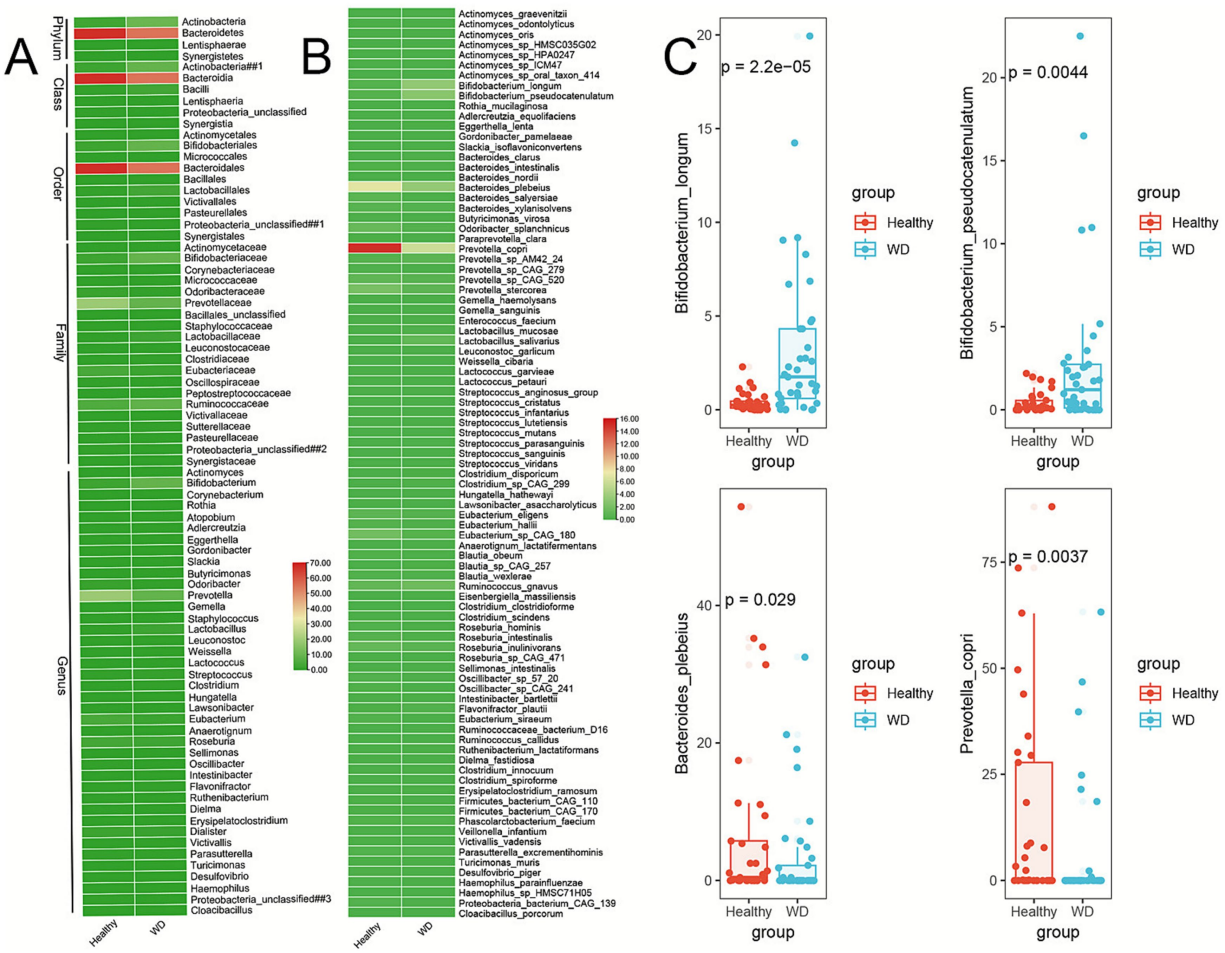
Taxonomic alterations in Wilson’s disease gut microbiota. **(A)** Heatmap of relative abundances at phylum to genus levels among Wilson’s disease and control groups. **(B)** Heatmap of relative abundances at species levels among Wilson’s disease and control groups. **(C)** Boxplots showing species significantly altered in WD vs. healthy controls (Wilcoxon test, *p* < 0.001). Example species: *Prevotella stercorea* (depleted), *Bifidobacterium longum* (enriched).

Bacterial profiles across various taxonomic levels were analyzed to characterize the gut microbial community structure in patients with WD and healthy controls (HCs) (Supplementary Figure S1). At the phylum level, a total of 10 phyla were identified, comprising 8 from the domain Bacteria, *Euryarchaeota from Archaea*, and *Ascomycota from Eukaryota*. *Actinobacteria, Bacteroidetes,* and *Firmicutes* were the predominant phyla in both groups, whereas *Lentisphaerae, Synergistetes,* and *Ascomycota* exhibited relative abundances slightly exceeding 1% (Supplementary Figure S1A; Supplementary Table S1). *Bacteroidia, Clostridia*, and *Actinobacteria* were the predominant classes, accounting for approximately two-thirds of the total microbial abundance (Supplementary Figure S1B; Supplementary Table S1). Significant differences in the relative abundances of *Actinobacteria, Synergistetes, Bacteroidetes,* and *Lentisphaerae* were observed across groups (*p* < 0.05, Figure 2A; Supplementary Table S1).

We further identified significant alterations in the relative abundance of 10 phyla, 21 classes, 34 orders, 63 families, 158 genera, and 436 species. Among these, 4 phyla, 6 classes, 10 orders, 20 families, 40 genera, and 89 species exhibited statistically significant differences between the WD and control groups (*p* < 0.05, Figure 2; Supplementary Table S1; Supplementary Figure S1). At the family level, *Lactobacillaceae*, *Ruminococcaceae*, and *Bifidobacteriaceae* were significantly enriched while *Prevotellaceae*, *Eubacteriaceae*, and *Odoribacteraceae* were depleted in the WD group compared to the control group (*p* < 0.05, Figure 2; Supplementary Table S1). These findings demonstrate that WD is associated with significant alterations in the gut microbial community.

**Figure 3:**
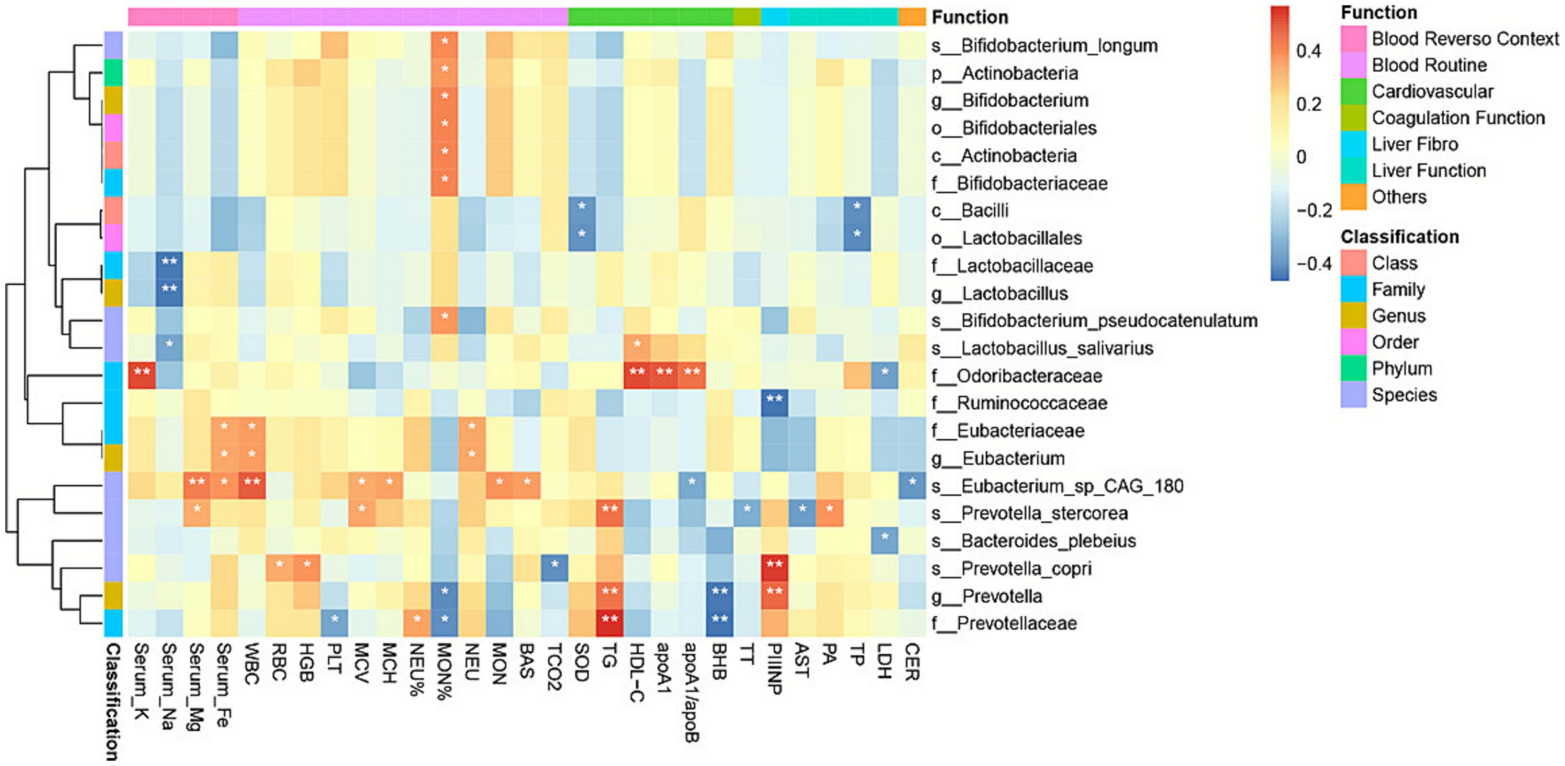
Microbial-clinical correlations in Wilson’s disease. Heatmap of Spearman correlations between microbial taxa (phylum to species) and clinical indicators. Key associations: *Prevotella* spp. positively correlated with PIIINP (liver fibrosis) and TG (cardiovascular risk); *Odoribacteraceae* positively correlated with HDL-C/apoA1 (cardioprotective markers); *Bifidobacterium* spp. associated with lipid metabolism markers. Color scale: red = positive correlation, blue = negative correlation.

Furthermore, we compiled a comprehensive set of up to 87 quantitative clinical indicators for patients with WD and examined their associations with gut bacterial abundance (Figure 3; Supplementary Figure S2; Supplementary Table S2). The abundance of the genus *Prevotella* and the species *Prevotella copri* showed a significant correlation with procollagen III N-terminal peptide (PIIINP), a biomarker closely associated with the activity of liver fibrosis (Członkowska et al., 2018). We also observed significant associations between multiple cardiovascular disease biomarkers and the abundance of specific microbial taxa. Specifically, s*__Prevotella_stercorea*, g*__Prevotella*, and f*__Prevotellaceae* exhibited strong correlations with triglycerides (TG), an established independent risk factor for coronary heart disease (Laufs et al., 2020). Furthermore, *Odoribacteraceae* was significantly associated with HDL−C, apoA1, and the apoA1/apoB ratio (von Eckardstein et al., 2023).

### Network analysis identifies species clusters with co-occurrence patterns

**Figure 4:**
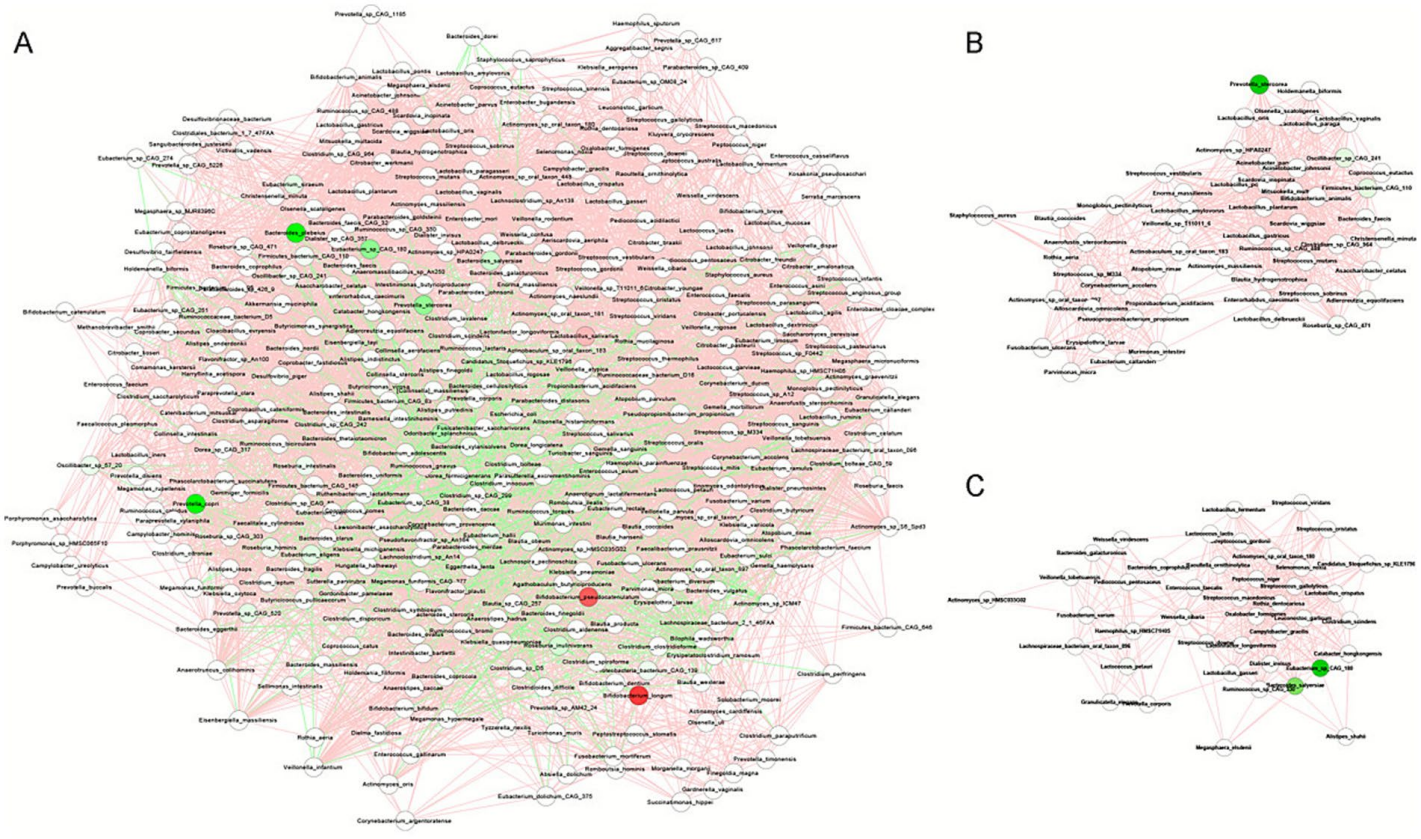
Gut microbial co-occurrence networks in Wilson’s disease. **(A)** Full co-occurrence network for WD patients (nodes = species; edges = significant positive correlations, Spearman |*r*| > 0.2, *p* < 0.05). **(B)** Cluster 1: Subnetwork of 48 species depleted in WD, including key taxa: *Prevotella stercorea*, *Firmicutes bacterium* CAG 110, *Oscillibacter* sp. CAG 241, *Roseburia* sp. CAG_471, *Adlercreutzia equolifaciens*, *Actinomyces* sp. HPA0247, and *Streptococcus mutans*. **(C)** Cluster 2: Subnetwork of 36 species depleted in WD, including key taxa: *Eubacterium* sp. CAG_180, *Bacteroides salyersiae*, *Lactococcus petauri*, *Leuconostoc garlicum*, *Haemophilus* sp. HMSC71H05, *Weissella cibaria*, *Clostridium scindens*, *Streptococcus cristatus*, *Actinomyces* sp. HMSC035G02, and *Streptococcus viridans*.

We conducted a comprehensive analysis of abundance correlations among 436 microbial species (Supplementary Table S3), revealing both co-occurring and competitive interactions that were utilized for network construction. Clustering analysis was performed using the MCODE plugin in Cytoscape. In total, 6,117 and 6,126 species-species interaction pairs were identified in the healthy control group and the WD group, respectively (Supplementary Table S3). A total of 808 species-species interaction pairs were identified as shared between the healthy control group and the WD group, indicating the presence of 5,308 health-associated specific pairs (Supplementary Figure S3) and 5,317 WD-specific pairs (Figure 4A). Furthermore, we conducted an in-depth analysis of species-species interactions within the WD group, focusing on the clusters identified by MCODE (Figure 4).

Cluster #1 contained 54 microbial species, of which seven (*Prevotella stercorea, Firmicutes bacterium* CAG 110, *Oscillibacter* sp. CAG 241, *Roseburia* sp. CAG_471, *Adlercreutzia equolifaciens, Actinomyces* sp. HPA0247, and *Streptococcus mutans*) exhibited reduced abundance in patients with WD. All of these species were interconnected through positive correlations. Notably, *P. stercorea* is a well-characterized bacterial strain that may play a key role in the gut microbial community of healthy individuals. Moreover, vitamin A and *β*-carotene, which are present in fruits such as bananas and mangoes, have been shown to enhance glucose metabolism by promoting the proliferation of *P. copri* and *P. stercorea;* however, these nutrients are significantly depleted in WD patients (Kovatcheva-Datchary et al., 2015; Pasolli et al., 2017) (Figure 4B).

Cluster #2 comprised 42 microbial species, among which six (*Eubacterium* sp. CAG_180, *Bacteroides salyersiae*, *Lactococcus petauri*, *Leuconostoc gelidum*, *Haemophilus* sp. HMSC71H05, and *Weissella cibaria*) exhibited decreased abundance in patients with WD. All of these species were interconnected through positive correlations (Figure 4B).

### Functional gene families linked to WD microbial shifts

Functional profiling using HUMAnN3 mapped metagenomic sequences to UniRef databases identified a total of 9,080 GO and 5,861 KO categories. Following normalization to copies per million reads (CPM), we observed 470 enriched (216 depleted) GO and 364 enriched (74 depleted) KO categories in patients with WD (*p* < 0.05). Among the most significantly enriched GO terms were GO:0046316 (gluconokinase activity), GO:0019546 (arginine deiminase pathway), GO:0047769 (arogenate dehydratase activity), GO:0008195 (phosphatidate phosphatase activity), and GO:0004133 (glycogen debranching enzyme activity), all of which showed a false discovery rate (FDR) < 0.05.

**Figure 5:**
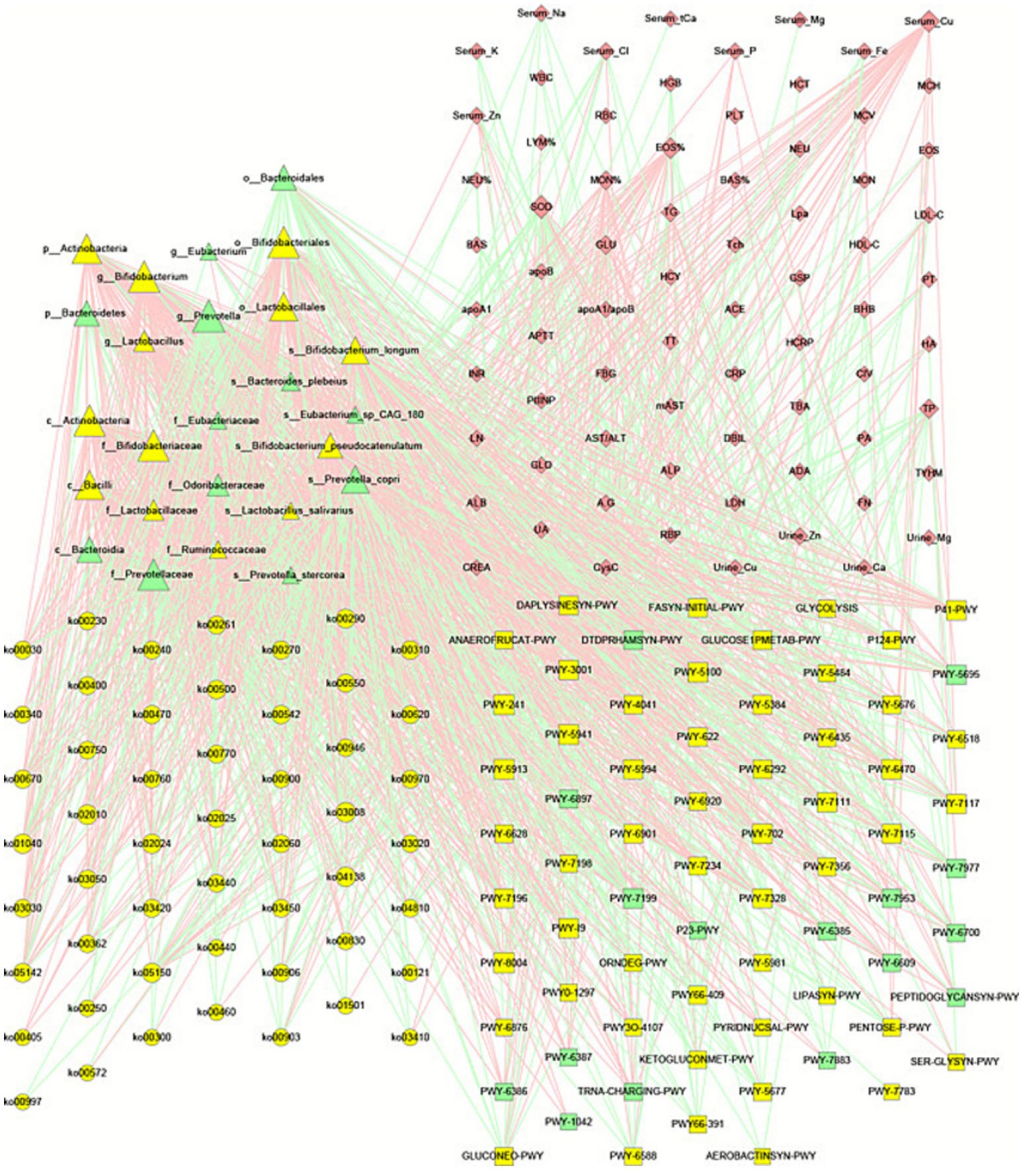
Correlation network of KEGG/MetaCyc pathways with clinical indicators and microbial taxa in Wilson’s disease. Network displays Spearman correlations between significantly altered metabolic pathways (KEGG ko-terms and MetaCyc PWY-terms), clinical parameters (e.g., liver function markers AST/ALT, DBIL; coagulation markers TT, FBG; lipid profiles HDL-C, TG), and microbial taxa (from phylum to species level).

We mapped KO categories to KEGG pathways by grouping functionally related KOs. Subsequently, differences between unstratified and stratified KO/KEGG categories were analyzed using the Wilcoxon rank-sum test. Notably, all 51 significantly differentially enriched KEGG terms were upregulated in patients with WD (*p* < 0.05, Supplementary Table S4), including ko00542 (O-Antigen repeat unit biosynthesis), ko00903 (Limonene degradation), and ko04138 (Autophagy - yeast) (Figure 5). Moreover, several KEGG terms corresponded to specific KO entries; for example, the enrichment of ko00542 may be attributed to the increased abundance of K02851 (UDP-GlcNAc undecaprenyl-phosphate/decaprenyl-phosphate GlcNAc-1-phosphate transferase, wecA, from *Bifidobacterium longum*) and K01354 (oligopeptidase B, ptrB, from *Bifidobacterium pseudocatenulatum*).

Specifically, the L-lysine biosynthesis I (DAPLYSINESYN-PWY), the superpathway of fatty acid biosynthesis initiation (*Escherichia coli*) (FASYN-INITIAL-PWY), Bifidobacterium shunt (P124-PWY), C4 photosynthetic carbon assimilation cycle, NADP-ME type (PWY-241), the superpathway of L-isoleucine biosynthesis I (PWY-3001), the *γ*-glutamyl cycle (PWY-4041), starch biosynthesis (PWY-622), pyruvate fermentation to isobutanol (engineered) (PWY-7111), the C4 photosynthetic carbon assimilation cycle, PEPCK type (PWY-7117), and the thiamine diphosphate salvage IV (yeast) (PWY-7356) were enriched in patients with WD. In contrast, pyrimidine deoxyribonucleosides salvage (PWY-7199) and creatinine degradation I (CRNFORCAT-PWY) showed enrichment in the control group, as shown in Figure 5, Supplementary Figure S4, and Supplementary Table S4.

To elucidate the associations between KEGG/MetaCyc pathway enrichment and microbial abundance, we employed the Spearman correlation coefficient to assess these relationships, as well as the correlations between KEGG/MetaCyc pathway enrichment and clinical indicators. Certain bacterial taxa were associated with GO/KO terms depleted in WD; specifically, *B. longum* and *B. pseudocatenulatum* exhibited strong positive correlations with the majority of WD-associated KEGG/MetaCyc categories, including ko00542 (O-Antigen repeat unit biosynthesis) and FASYN-INITIAL-PWY (superpathway of fatty acid biosynthesis initiation). In contrast, these taxa showed strong negative correlations with several MetaCyc pathways, such as DTDPRHAMSYN-PWY (dTDP-beta-L-rhamnose biosynthesis) and inosine 5′-phosphate degradation (Figure 5).

### Disruption of CAZy in WD

**Figure 6:**
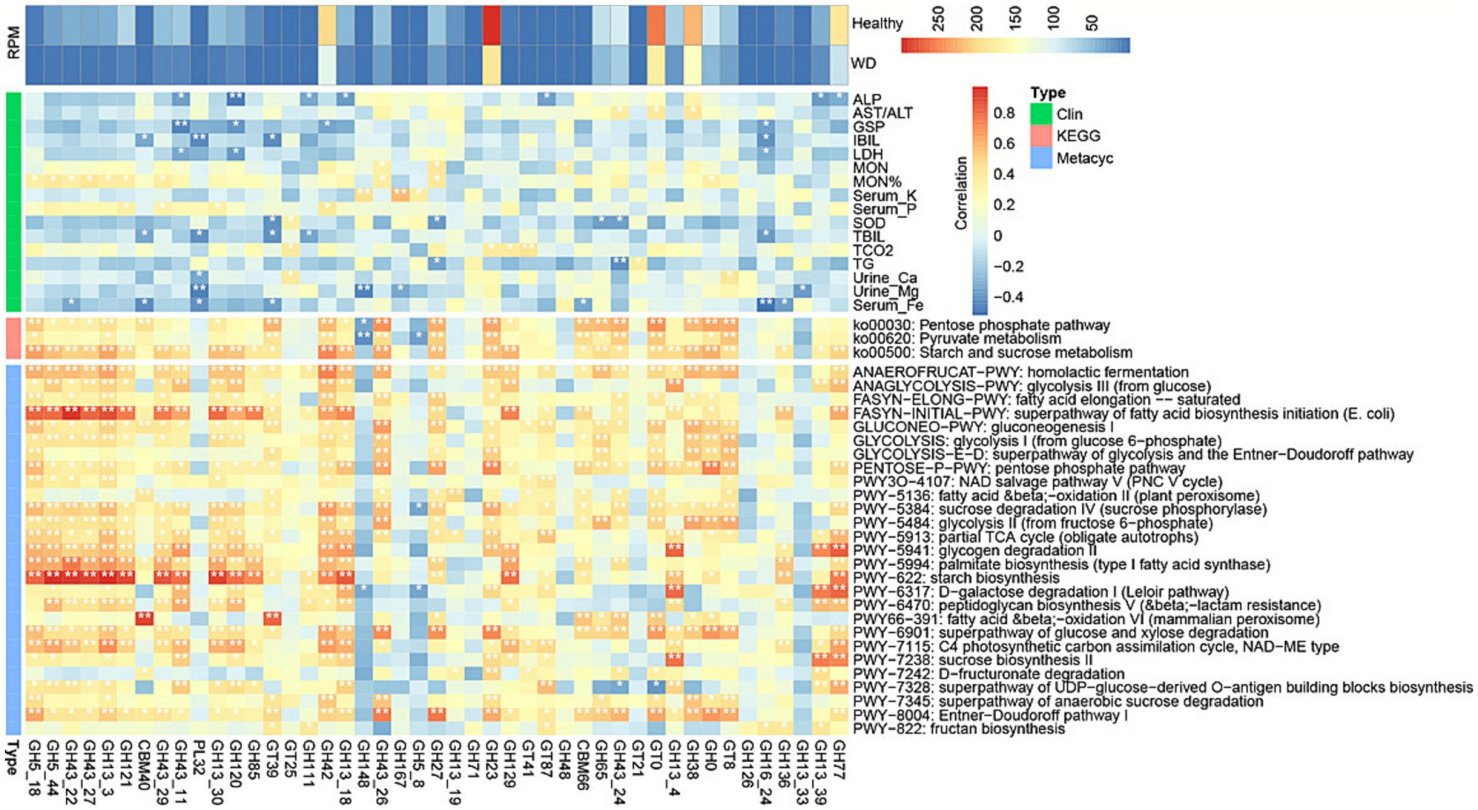
Correlations between CAZy genes and metabolic pathways in WD. Heatmap showing spearman correlations between differentially abundant CAZy genes (GHs, CBMs, GTs) and carbohydrate metabolic pathways in Wilson disease (WD) patients versus healthy controls.

CAZy enzymes, which are essential for carbohydrate degradation, significantly impact both gut microbiota and the host. The functional analysis showed that carbohydrate-related pathways were significantly different, including the Pentose phosphate pathway (ko00030), Pyruvate metabolism (ko00620), Starch and sucrose metabolism (ko00500), ANAEROBIC GLYCOLYSIS−PWY: glycolysis III (from glucose), fatty acid elongation saturated (FASYN−ELONG−PWY), and the superpathway of fatty acid biosynthesis initiation (*E. coli*) (FASYN−INITIAL−PWY) (Supplementary Table S4 and Supplementary Figure S5). Therefore, we employed the CAZy database to functionally annotate metagenomic data in order to investigate their potential roles. CAZy-based annotation identified two downregulated genes (PL21, GT72) and 13 upregulated genes (GH5_18, GH5_44, GH43_27, GH43_22, GH13_3, GH121, GH43_11, GH43_29, GH13_30, GH120, CBM40, GH85, GT71) in patients with WD (Figure 6; Supplementary Table S5). We found that almost all of the 13 upregulated genes belong to glycoside hydrolases (GHs), excluding one carbohydrate-binding module (CBM40) and one glycosyl transferase (GT71). Furthermore, we compared CAZy gene distributions at the species level and constructed a CAZy-species co-occurrence network. Analysis revealed that Bifidobacterium exhibited the highest abundance levels of CAZy genes, a result consistent with the patterns observed in the CAZy-species co-occurrence network. Collectively, these findings suggest that probiotics may modulate CAZy enzyme activity in the context of WD.

Furthermore, our analysis revealed that several GHs, including GH77, GH42, GH120, GH121, and GH85, exhibited strong positive correlations with taxonomic groups at multiple levels, including the class *Actinobacteria*; the family *Bifidobacteriaceae*; the order *Bifidobacteriales*; the genus *Bifidobacterium*; and the species *B. longum* and *B. pseudocatenulatum*. Moreover, these GHs also showed significant positive correlations with the order *Bacteroidales*, the species *Bacteroides plebeius*, and the class *Bacteroidia*. Additionally, our analysis revealed that several GHs, including GH38, GH27, and GT0, along with CBM40 and GT39, exhibited strong positive correlations with the genus *Prevotella*, the species *P. copri*, the species *P. stercorea*, and the family *Prevotellaceae*.

### VF gene composition distinguishes between WD and control

Initially, we identified multiple gene sequences within the metagenomic data exhibiting high similarity to known virulence factor genes in the VFDB database. These findings indicate the potential presence of virulence factors in the analyzed samples.

A total of 54 WD-specific Virulence Factor-Encoding Genes (VFGs) genes were identified through DESeq2 (read counts) and Wilcoxon tests (RPM), with 36 genes showing significant depletion and 18 exhibiting significant enrichment. These differentially expressed genes included 1 from *Enterococcus faecalis*, 22 from *E. coli*, 12 from *Haemophilus influenzae*, 5 from *Klebsiella pneumoniae*, 3 from *S. mutans*, 2 from *Streptococcus sanguinis*, and 2 from *Mycobacterium tuberculosis* (Supplementary Figure S6). A total of eight colibactin genes exhibited significant depletion: *clbB*, *clbH*, *clbJ*, and *clbK* from *E. coli*, along with *clbG*, *clbI*, *clbO*, and *clbP* from *K. pneumoniae*. Furthermore, seven type IV secretion system genes: *aec19*, *aec29*, *aec31*, *clbB*, *clbK*, *hcp*, and *ehaB*—derived from *E. coli* showed reduced abundance in WD, whereas *pilB* from *H. influenzae* was significantly enriched in WD.

**Figure 7:**
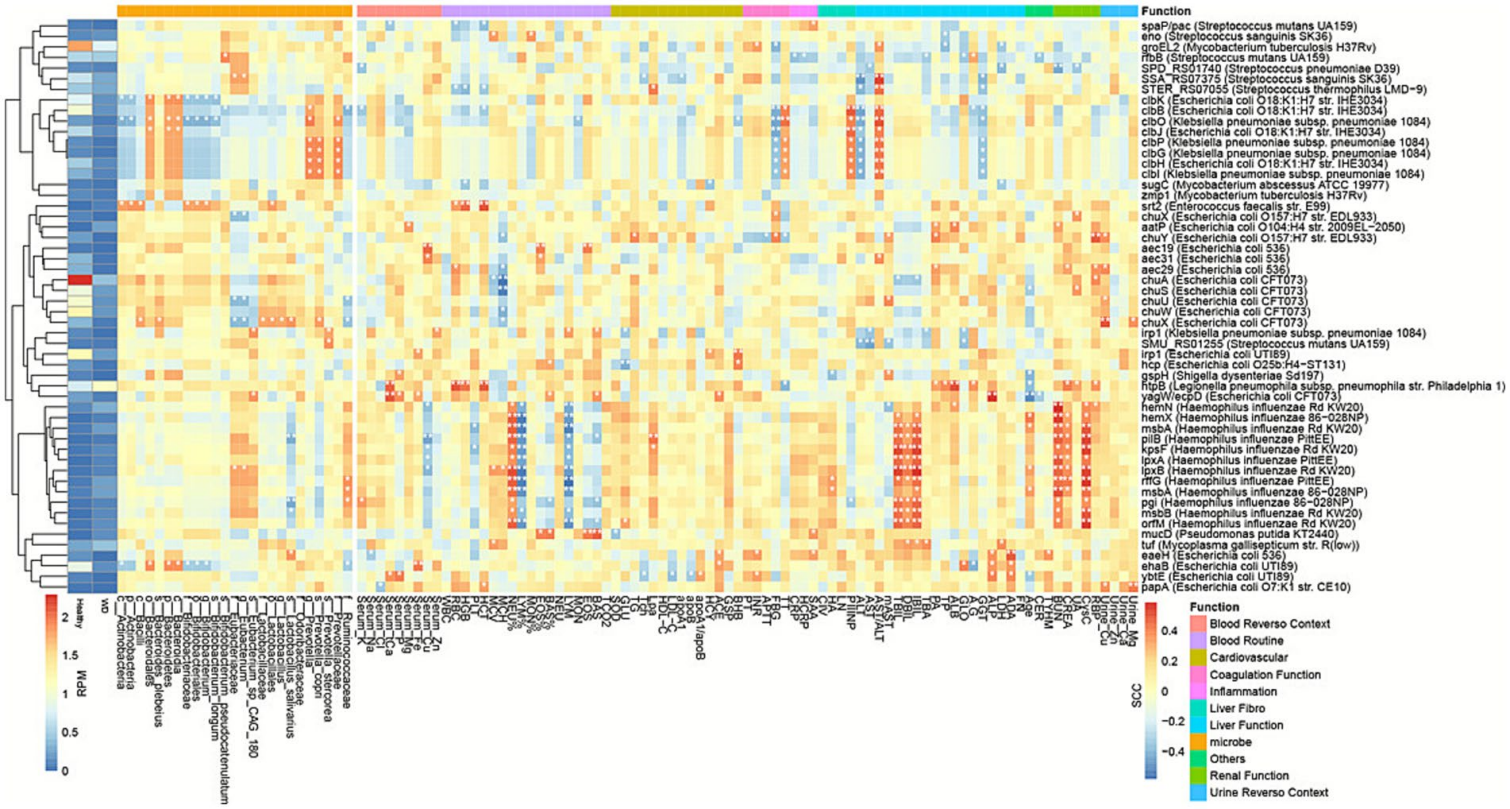
Virulence factor associations with microbiota and clinical indicators in Wilson’s disease. Heatmap displays Spearman correlations between differentially abundant virulence factors (VFs), microbial taxa, and clinical parameters (*p* < 0.05).

Furthermore, our analysis identified the presence of virulence factors associated with other pathogens. *ehaB* and *clbK* from *E. coli*, together with *clbO* from *K. pneumoniae,* showed associations with the taxa *Actinobacteria*, *Bacteroidales*, and *Bifidobacterium*. Additionally, colibactin genes, including *clbO*, *clbP*, *clbG*, and *clbI* from *K. pneumoniae* as well as *clbH* from *E. coli*, were found to be associated with *Prevotella* (Figure 7). These findings suggest the existence of diverse pathogenic organisms within the metagenomic dataset, each with the potential to exert distinct virulence effects on the host.

We also employed the Spearman correlation coefficient to assess the correlations and associations between virulence factors (VFs) and clinical indicators. *hemX*, *msbA*, *pilB*, *kpsF*, *lpxA*, *lpxB*, *rffG*, *pgi*, *msbB*, and *orfM* from *H. influenzae* were significantly correlated with TBIL (Total Bilirubin), DBIL (Direct Bilirubin), and IBIL (Indirect Bilirubin), which are established markers of liver function (Lindor et al., 1979; Lester and Schmid, 1964). Moreover, these VFs demonstrated associations with routine blood parameters, including Neutrophil Percentage (NEU%), Lymphocyte Percentage (LYM%), and Lymphocyte Count (LYM). In parallel, colibactin genes such as *clbO*, *clbP*, *clbG*, and *clbI* from *K. pneumoniae,* along with *clbH* from *E. coli*, were linked to coagulation function [Fibrinogen (FBG) and Thrombin Time (TT)], liver fibrosis (PIIINP), and hepatic function (AST and AST/ALT) (Figure 7).

In summary, the analysis of metagenomic data integrated with the VFDB database identifies multiple potential virulence factors present in our samples. These virulence factors are linked to diverse pathogens and may exert distinct pathogenic effects on the host. Our findings contribute to a deeper understanding of pathogenic virulence mechanisms and offer novel insights for the development of therapeutic strategies targeting WD.

### Resistance markers are differentially abundant with WD

**Figure 8:**
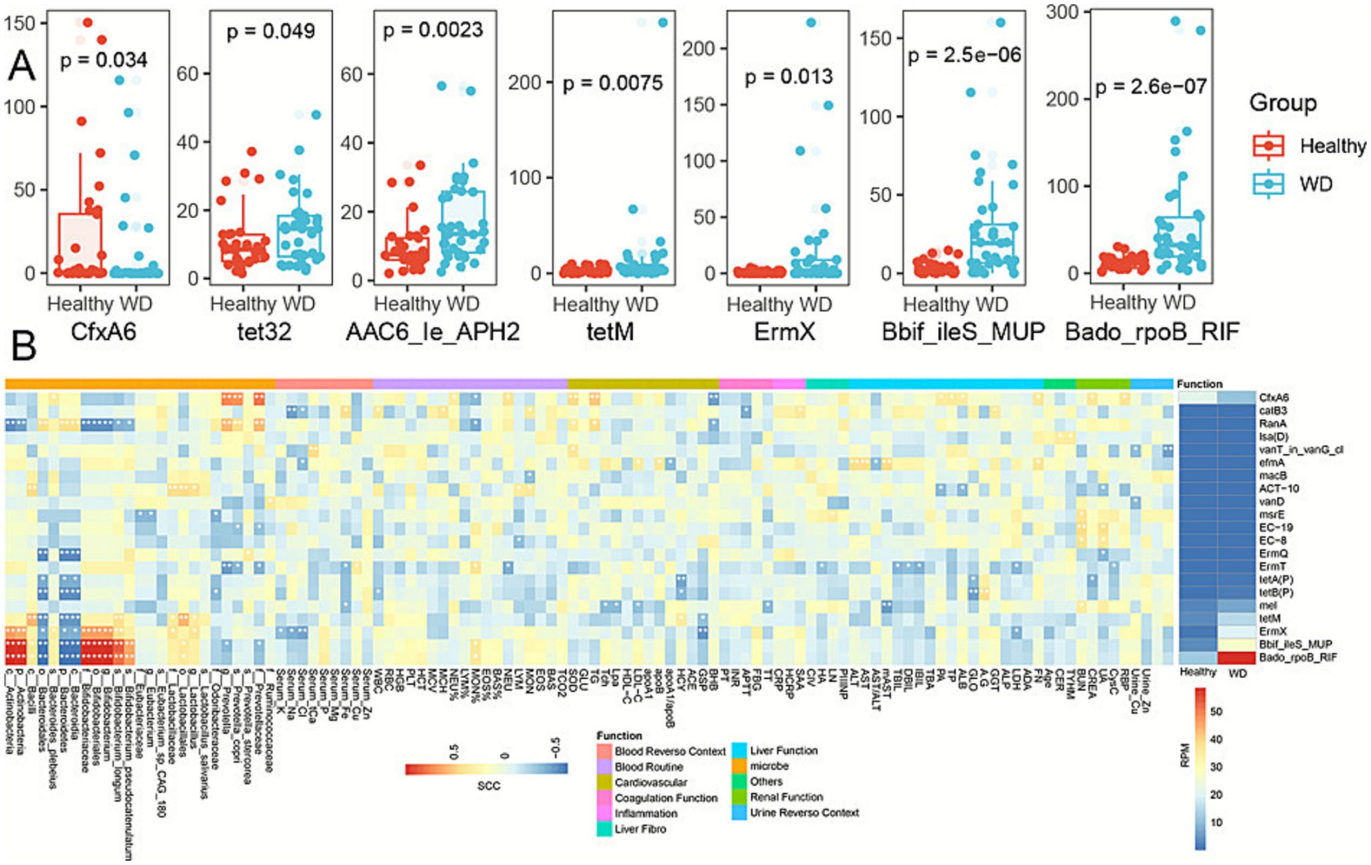
Antibiotic resistance gene profiles in Wilson’s disease. **(A)** Differential abundance of key ARGs. Boxplots show RPM distribution of seven significantly altered antibiotic resistance genes in healthy controls vs. Wilson’s disease (WD) patients. Significance by Wilcoxon test. **(B)** Correlation network with microbiota and clinical parameters. Heatmap displays Spearman correlations between ARGs and gut microbiota/clinical indicators.

We characterized the gut resistome composition in individuals with WD and HCs through an analysis of CARD-mapped reads. Notable ARGs identified include tetracycline-resistant ribosomal protection proteins [*tetQ* (Tribble et al., 2010; Leng et al., 1997), *tetO* (Luna and Roberts, 1998; LeBlanc et al., 1988), and tetW (Scott et al., 2000); conferring resistance to tetracycline], rm family genes encoding 23S ribosomal RNA methyltransferases [*ermB* (Min et al., 2008) and *ermF* (Kangaba et al., 2015); mediating resistance to streptogramin, macrolide, and lincosamide], *cfxA beta-lactamase genes* [*cfxA2* (Madinier et al., 2001) and c*fxA6* (Sommer et al., 2009); conferring resistance to cephamycin], as well as *Bado_rpoB_RIF*, *Mef (En2)*, and *Bbif_ileS_MUP*. Our analysis revealed 21 ARGs with statistically significant differential abundance in WD compared to the control group, as determined by integrated analysis using the Wilcoxon test with RPM and DESeq2 count data (*p* < 0.05) (Figure 8; Supplementary Figure S7; Supplementary Table S6). Among these differentially represented ARGs was the *Bifidobacterium bifidum ileS* gene conferring resistance to mupirocin (*Bbif_ileS_MUP*) (Serafini et al., 2011), which is predominantly encoded by *Bifidobacterium* species and mediates resistance to mupirocin. Furthermore, *Bifidobacteria* exhibit intrinsic resistance mechanisms that confer resistance to rifampicin, represented by the gene *Bado_rpoB_RIF* (Lokesh et al., 2018).

We evaluated the associations between microbial taxa and ARGs through Spearman correlation analysis. The class A *β*-lactamase gene *cfxA6* (Sommer et al., 2009), exhibited significant depletion in individuals with WD, as observed for the genus *Prevotella,* species *P. copri*, and family *Prevotellaceae*. *Bbif_ileS_MUP*, *Bado_rpoB_RIF*, and *ermX* (a ribosomal methyltransferase conferring antibiotic resistance) (Rosato et al., 2001), were significantly enriched in individuals with WD, displaying a consistent correlation pattern with microbial taxa. These ARGs showed significant positive correlations with the class *Actinobacteria*, family *Bifidobacteriaceae*, order *Bifidobacteriales*, genus *Bifidobacterium*, species *B. longum*, and species *B. pseudocatenulatum.* Conversely, they exhibited significant negative correlations with the order *Bacteroidales*, species *B. plebeius*, and class *Bacteroidia.* Additionally, the associations between ARGs and clinical indicators were assessed through Spearman correlation analysis. MsrA homolog, an ABC-F family protein associated with macrolide resistance (Chouchani et al., 2012), was significantly enriched in individuals with WD. Moreover, it exhibited significant positive correlations with serum biomarkers including Mitochondrial Aspartate Aminotransferase (mAST), TG, Total Cholesterol (Tch), Lipoprotein (a) (Lpa), High-Density Lipoprotein Cholesterol (HDL−C), and Low-Density Lipoprotein Cholesterol (LDL−C) (Figure 8).

### WD-associated enrichment of functional Mobile genetic elements

**Figure 9:**
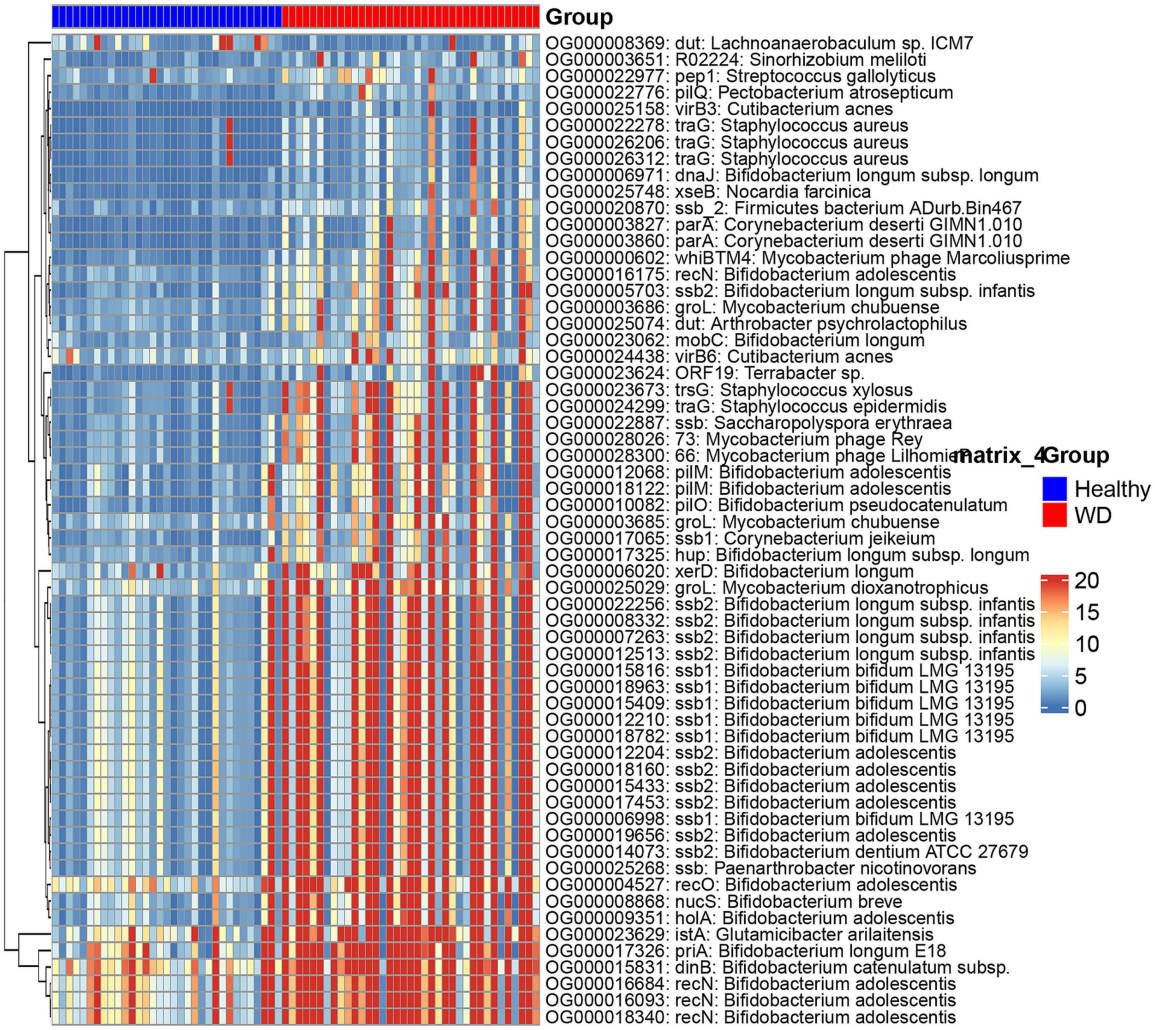
Different abundance profile of MGEs across WD and healthy groups. Clustered heatmap of MGE abundance (RPM) in individual samples.

Comparative metagenomic analysis using rigorous statistical testing identified a total of 60 mobile genetic elements (MGEs) exhibiting a statistically significant differential abundance between the WD cohort and healthy controls (Supplementary Table S7; Figure 9). Strikingly, the vast majority of these elements demonstrated a marked and significant increase in their abundance within the disease state microbiome. This widespread upregulation strongly indicates a pronounced activation of the mobile gene pool, highlighting a key distinction in the genetic architecture of the microbial community associated with the disease.

Functional categorization of these differential MGEs reveals a coherent enrichment in four primary biological processes fundamental to genetic mobility and adaptation. The most prominent category comprises MGEs involved in DNA replication, recombination, and repair, including elements encoding functions such as RecN, DinB, PriA, and NucS. This suggests an environment of genomic stress or instability. A substantial fraction consists of phage-derived MGEs, carrying genes for single-stranded DNA-binding proteins (e.g., SSb) and regulatory proteins like WhiBTM4, pointing to increased phage activity and potential for lysogenic conversion. Critically, MGEs essential for conjugative transfer—encoding apparatus components such as TraG, VirB proteins, and type IV pilus assembly proteins—are significantly enriched, signaling an enhanced capacity for horizontal gene transfer via bacterial conjugation. A final set of MGEs is linked to DNA integration/excision and regulatory functions, including tyrosine recombinases (XerD), transposases, and mobilization regulators.

These differentially abundant MGEs originate from key bacterial genera, most notably Bifidobacterium, Mycobacterium, Staphylococcus, and Corynebacterium. The collective enrichment of these functional MGE suites paints a picture of a disease-state microbiome characterized by heightened genomic plasticity. The concurrent upregulation of phage-related and conjugation-associated elements implies a synergistic increase in the major pathways for horizontal gene transfer. This environment likely fosters the rapid dissemination and acquisition of adaptive genetic traits, such as antimicrobial resistance or virulence factors, within the microbial consortium. Consequently, the dynamics of MGE activation and transfer appear to be a central feature of the dysbiotic microbiome, potentially playing a direct contributory role in disease pathophysiology.

### Correlational network analysis reveals strong and specific linkages between antibiotic resistance genes and functional Mobile genetic elements

**Figure 10:**
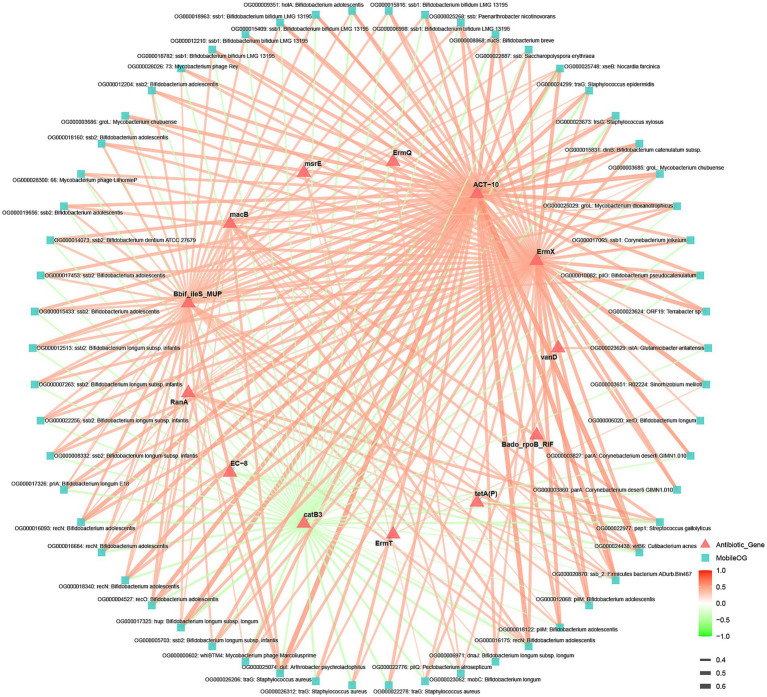
Correlation network of ARGs and MGEs. Correlations shown are significant (Spearman’s *r* > 0.3, *p* < 0.05).

Based on an analysis of statistically significant positive correlations (*p* < 0.05, Spearman’s *ρ* ≥ 0.3) between antibiotic resistance genes (ARGs) and mobile genetic elements (MGEs), distinct patterns of co-occurrence were identified, suggesting specific genetic vehicles for resistance dissemination. (Supplementary Table S8; Figure 10).

The erythromycin resistance gene ErmX demonstrated the broadest and strongest network of associations. Its most significant correlation was with a transposase (ORF19 from Terrabacter sp., ρ = 0.535, *p* = 6.71e–40). Furthermore, ErmX showed consistently strong correlations (*ρ* ~ 0.43–0.51) with MGEs spanning multiple functional categories: (1) Phage-related replication proteins (e.g., various Ssb proteins from Bifidobacterium spp., Corynebacterium, and Mycobacterium phages), (2) DNA repair/recombination proteins (e.g., RecN, RecO, DinB, PriA, XseB), (3) Conjugation apparatus components (e.g., TraG/TrsG from Staphylococcus, PilM, PilO), and (4) Chaperones/regulators (GroL, Dut). This pattern positions ErmX as a highly mobile resistance determinant potentially linked to diverse genetic platforms, including phages, conjugative elements, and transposons.

The aminoglycoside resistance gene ACT-10 showed exceptionally strong, specific correlations with conjugative transfer proteins, particularly TraG variants from *Staphylococcus aureus* (*ρ* > 0.46), highlighting a probable primary association with staphylococcal conjugative systems. Other ARGs, including Bbif_ileS_MUP, macB, and RanA, also showed significant positive correlations, primarily with the Ssb and Rec protein families from Bifidobacterium, indicating a potential common reservoir or co-mobilization niche within this genus.

Functionally, the MGEs forming hubs in this network fall into three key groups: (1) Replication/Stability (phage/plasmid SSb proteins, DNA repair enzymes), (2) Horizontal Transfer (conjugation ATPases, pilus assembly proteins), and (3) Integration/Mobilization (transposases, relaxosome proteins). The prevalence of MGEs originating from Bifidobacterium and Mycobacterium species is notable, suggesting these taxa may be important reservoirs or vectors for the identified ARGs.

In summary, correlation network analysis reveals that specific ARGs, most prominently ErmX, are strongly co-associated with defined suites of MGEs. These associations point to plausible mechanistic links: ErmX may be frequently encoded on genetic elements that utilize Bifidobacterium-derived phage-like replication modules (ssb, rec) and are mobilized by staphylococcal conjugation systems (traG) or transposition (ORF19). These prioritized ARG-MGE pairs represent high-confidence candidates for future investigations into physical linkage (e.g., via contig analysis or PCR) and the mechanistic basis of resistance spread.

## Discussion

This study provides a comprehensive metagenomic characterization of the gut microbiome in WD, extending beyond the limitations of prior 16S rRNA sequencing studies (Pu et al., 2025; Tang et al., 2025; Geng et al., 2018). We identified species-level microbial signatures exhibiting significant enrichment or depletion in WD, which were also strongly associated with clinical indicators. Furthermore, a microbial co-occurrence network was constructed to elucidate potential inter-taxa relationships. Functional analysis (including GO, KO, Metacyc pathways, and KEGG pathways) revealed significant functional distinctions between individuals with WD and HCs. Multiple functional terms were found to correlate with microbiome markers and clinical indicators, indicating potential mechanistic roles. Notably, we identified three key functional alterations, including the disruption of carbohydrate metabolism (e.g., CAZymes GH5_18 and GH43_27 in starch and sucrose metabolic pathways). Alterations in VFs were observed, with 54 differentially abundant virulence-associated genes identified, including those within the colibactin biosynthetic clusters. Furthermore, significant enrichment of ARGs was detected, as exemplified by tetracycline-resistant *tetQ* and macrolide-resistant *ermB*.

We know that WD is characterized by dysregulated copper ion metabolism, leading to systemic organ involvement, particularly affecting the liver, brain, and kidney. Accumulating evidence has established associations between gut microbiota and disease pathogenesis through key microbial-host interaction pathways, including the gut-brain axis (Loh et al., 2024; Aburto and Cryan, 2024), gut-liver axis (Pabst et al., 2023; Hsu and Schnabl, 2023), and gut-kidney axis (Yang et al., 2018). This study observed alterations across the entire taxonomic spectrum. Our findings demonstrate that the species *B. longum* and *B. pseudocatenulatum* exhibit significantly higher abundance in individuals with WD compared to HCs. This pattern is consistent with their higher-level taxonomic classifications, including the order *Bifidobacteriales*, family *Bifidobacteriaceae*, and genus *Bifidobacterium*. Accumulating evidence suggests that members of the genus *Bifidobacterium* are associated with the progression of metabolic and neurological disorders, including non-alcoholic fatty liver disease (NAFLD) (Scorletti et al., 2020), hepatocellular carcinoma (Song et al., 2023), and neurological diseases such as Parkinson’s disease (Bolliri et al., 2022).

Our results further demonstrate that members of the genus *Prevotella*, including *P. copri* and *P. stercorea* from the family *Prevotellaceae*, exhibit dysbiosis in WD and show a positive association with liver fibrosis, in line with previous findings (Dubinkina et al., 2017; Pirola et al., 2022). Moreover, a marked reduction was observed in the primary symbiotic bacteria within the *Bacteroidetes* phylum (Dubinkina et al., 2017). Alterations in bile secretion and composition may account for the observed microbial changes, aligning with findings from microbiome studies in non-alcoholic cirrhosis (Qin et al., 2014). In our study, the species *B. plebeius*, belonging to the order *Bacteroidales*, exhibited significant dysregulation. The family *Ruminococcaceae* plays a crucial role in maintaining gut microbiota homeostasis (Qin et al., 2014). As previously reported (Zhu et al., 2013; Loomba et al., 2017; Bajaj et al., 2014), rumen-derived microbiota progressively decline in abundance as fibrosis severity increases, particularly in non-obese individuals with comorbid conditions and NAFLD. While studies conducted in Latin American populations with non-alcoholic steatohepatitis (NASH) and Asian cohorts with NAFLD have identified distinct microbiome profiles associated with obesity status (Duarte et al., 2018; Wang et al., 2016). Boursier et al. (2016) reported a significant increase in the abundance of *Ruminococcus* species among patients with advanced fibrosis. In our study, the family *Ruminococcaceae* demonstrated a significant reduction in abundance, which was strongly inversely correlated with the severity of liver fibrosis. Furthermore, an elevated abundance of the species *Lactobacillus salivarius* and its higher taxonomic groups—including the genus *Lactobacillus*, family *Lactobacillaceae*, and order *Lactobacillales*—was observed in association with WD.

Symbiotic bacteria, including members of the *phyla Bacteroidetes, Firmicutes, Actinobacteria*, and *Proteobacteria*, are likely to harbor the majority of ARGs in the intestines of healthy individuals, thereby serving as a potential reservoir for the transmission of ARGs to common intestinal pathogens. The colonization of symbiotic anaerobic bacteria harboring ARGs provides a plausible explanation for the detection of ARGs even in the absence of antibiotic selective pressure. Our findings indicate that the presence of ARGs contributes to resistance against multiple antibiotic classes, including tetracyclines, streptogramins, macrolides, lincosamides, cephamycins, and aminoglycosides, a pattern that is comparable to but distinct from previously reported results (Lokesh et al., 2018; Heston et al., 2024; Gibson et al., 2016), Although the study population exhibited minimal exposure to these antibiotic categories, notably, distinct differences in ARGs were observed between WD patients and HCs. The characterization of intestinal colonization by ARG-carrying bacteria is particularly relevant for WD patients with concomitant microbial dysbiosis. Our findings demonstrate significant associations between ARG profiles, microbial community composition, and clinical parameters in WD, indicating that modulation of the gut microbiota may offer a potential therapeutic strategy for symptom improvement in these patients.

Virulence factors represent critical attributes of potentially pathogenic bacteria that confer selective advantages over commensal members of the healthy gut microbiota. Changes in mucosal composition, enhanced bacterial adhesion, toxin production, and resource competition with the host (Sanchez-Villamil and Navarro-Garcia, 2015) may contribute to the pathogenic mechanisms underlying WD, with specific virulence factors associated with hepatic function and immune responses. To date, investigations into virulence mechanisms have predominantly centered on gastrointestinal disorders, particularly involving specific bacterial taxa such as *adherent-invasive E. coli* and microbial proteases (Vich Vila et al., 2018). Investigation into the pathogenic potential of the gut microbiota in WD may reveal additional therapeutic targets for patients affected by this condition. While these findings require further validation through targeted experimental approaches or transcriptomic profiling, the proposed association between virulence factors and disease mechanisms offers a foundation for elucidating the pathogenesis of WD.

This study has certain limitations. It is important to note that functional annotation databases, including CAZy, VFDB, and CARD, have inherent limitations such as reliance on known sequences, potential annotation update delays, and incomplete coverage of microbial diversity. These limitations may affect the completeness of our functional annotations and should be considered when interpreting the results. Minority bacterial populations may contribute to the pathogenesis of WD, however, current metagenomic methodologies may lack the sensitivity to detect these low-abundance taxa. Furthermore, fecal microbiota transplantation experiments could serve as a valuable approach for functional validation in future investigations.

## Data Availability

The original contributions presented in the study are included in the article/Supplementary material, further inquiries can be directed to the corresponding author.

## Supplementary material

SUPPLEMENTARY FIGURE S1 Gut microbial composition in WD versus healthy controls. **(A)** Phylum, class, and order levels: Bar plots show relative abundances (samples grouped: WD on top, controls below). WD exhibits increased Proteobacteria (orange) and decreased Firmicutes (blue); class Gammaproteobacteria and order Enterobacterales are enriched in WD. **(B)** Family and genus levels: Bifidobacteriaceae and Bifidobacterium (purple) are significantly enriched in WD, while Prevotellaceae (red) increases in select WD samples. Controls are dominated by Bacteroidaceae and Bacteroides (green). **(C)** Species level: *B. longum* and *B. pseudocatenulatum* are enriched in WD, whereas *B. vulgatus* and *F. prausnitzii* dominate controls. All levels indicate dysbiosis in WD.

SUPPLEMENTARY FIGURE S2 Correlations between gut microbiota and clinical parameters. Spearman correlations between gut microbiota (phylum to species) and clinical categories. Red: positive; blue: negative. Bifidobacterium positively correlates with HDL-C and negatively with IBIL; Prevotella positively correlates with liver function markers (ALT/AST) and negatively with serum zinc. Clinical indices grouped by function: Liver Function, Cardiovascular, etc.

SUPPLEMENTARY FIGURE S3 Species co-occurrence network in healthy controls. Gut microbial species co-occurrence network in healthy controls. Nodes represent species (colored by taxa), edges indicate significant positive co-occurrence. Key hub species include *Bifidobacterium longum*, *Prevotella copri*, and *Bacteroides vulgatus*. The high connectivity reflects stable ecological interactions in a healthy microbiome.

SUPPLEMENTARY FIGURE S4 Heatmap displays Spearman correlations between significantly altered metabolic pathways (KEGG ko-terms and MetaCyc PWY-terms), clinical parameters (e.g., liver function markers AST/ALT, DBIL; coagulation markers TT, FBG; lipid profiles HDL-C, TG), and microbial taxa (from phylum to species level).

SUPPLEMENTARY FIGURE S5 Integrated associations of CAZy genes with microbiota, metabolic pathways, and clinical indices in WD. **(A)** Correlations between CAZy genes and microbiota/clinical indices: Spearman correlations (|r| > 0.4, FDR < 0.05) between differentially abundant CAZy genes and gut microbiota (genus/species level) or clinical indices. **(B)** Associations of CAZy genes with MetaCyc pathways: Spearman correlations between CAZy genes and MetaCyc metabolic pathways. **(C)** Associations of CAZy genes with KEGG pathways: Spearman correlations between CAZy genes and KEGG carbohydrate metabolism pathways.

SUPPLEMENTARY FIGURE S6 Differential abundance of virulence factor (VF) genes in the gut of WD patients versus controls. Heatmap shows 54 differentially expressed VF genes identified by DESeq2 (read counts) and Wilcoxon test (RPM).

SUPPLEMENTARY FIGURE S7 ARG abundance profile across sample groups. Clustered heatmap of ARG abundance (RPM) in individual samples. Columns represent samples grouped by Healthy (green) and WD (orange); rows represent ARGs.

SUPPLEMENTARY TABLE S1 Taxonomic abundance at different levels and statistical comparisons.

SUPPLEMENTARY TABLE S2 Clinical indicators and their correlations with microbial taxa.

SUPPLEMENTARY TABLE S3 Species-species interaction pairs in WD and control groups.

SUPPLEMENTARY TABLE S4 Functional pathway enrichment results.

SUPPLEMENTARY TABLE S5 CAZy gene abundance and statistical analysis.

SUPPLEMENTARY TABLE S6 ARGs abundance and statistical analysis.

SUPPLEMENTARY TABLE S7 MGEs abundance and statistical analysis.

SUPPLEMENTARY TABLE S8 Spearman correlation analysis between ARGs and MGEs.
