# Supplementary figures and images for: Wilson’s disease-associated gut dysbiosis: novel insights into microbial functional alterations, virulence changes, and resistance markers
Source: Front Microbiol. 2026 Jan 15;16:1714276. doi: 10.3389/fmicb.2025.1714276 (PMC12853645; doi:10.3389/fmicb.2025.1714276)

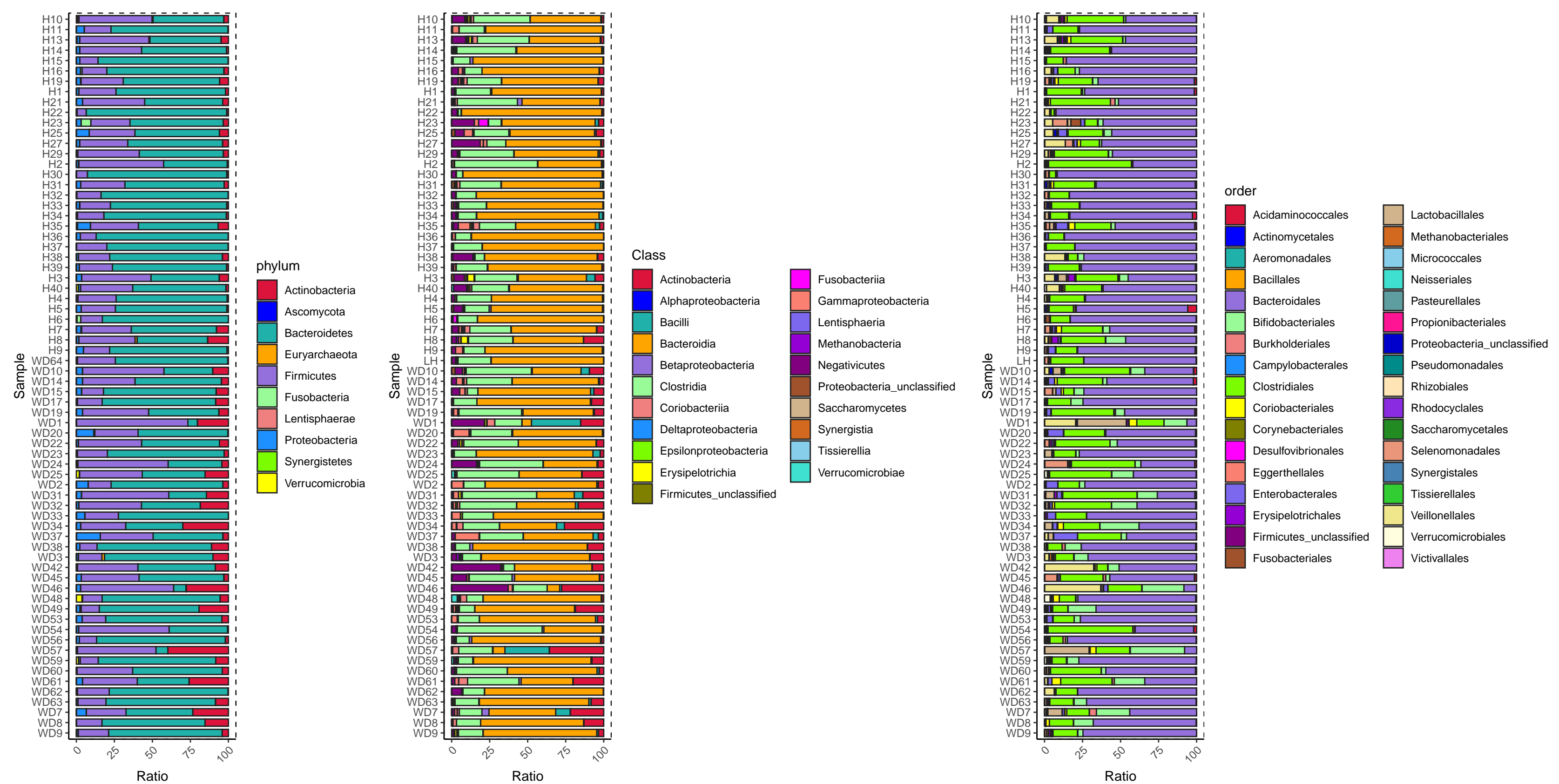

Supplement: Supplementary file 1 [file Data_Sheet_1.PDF]

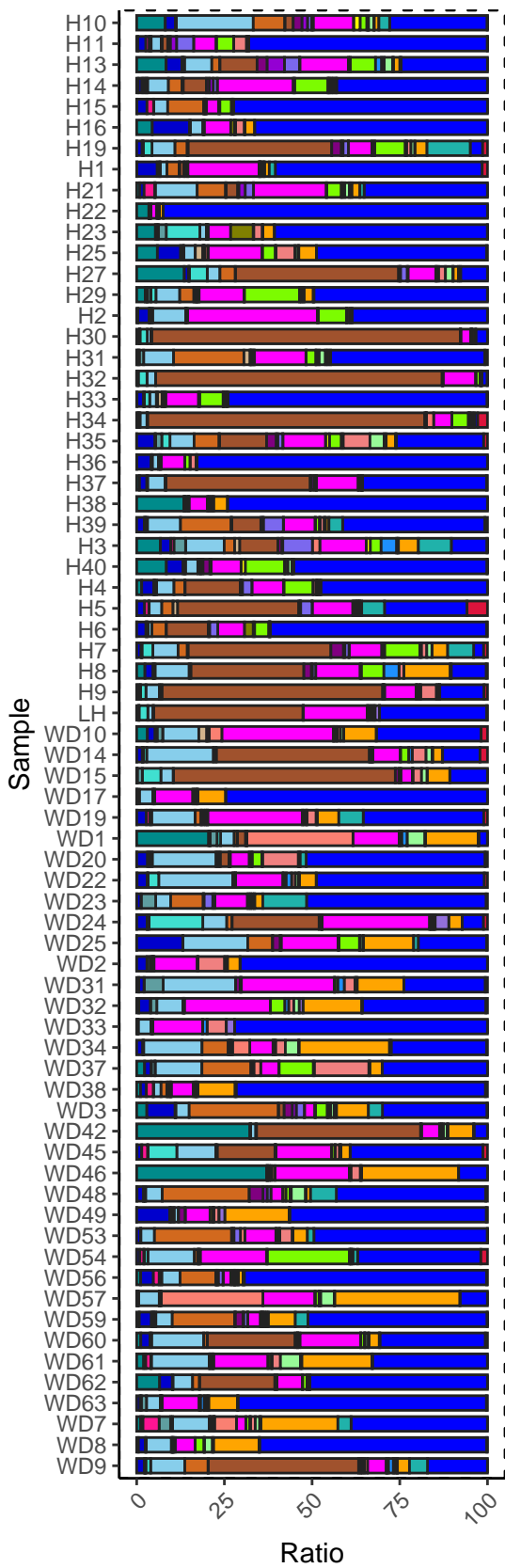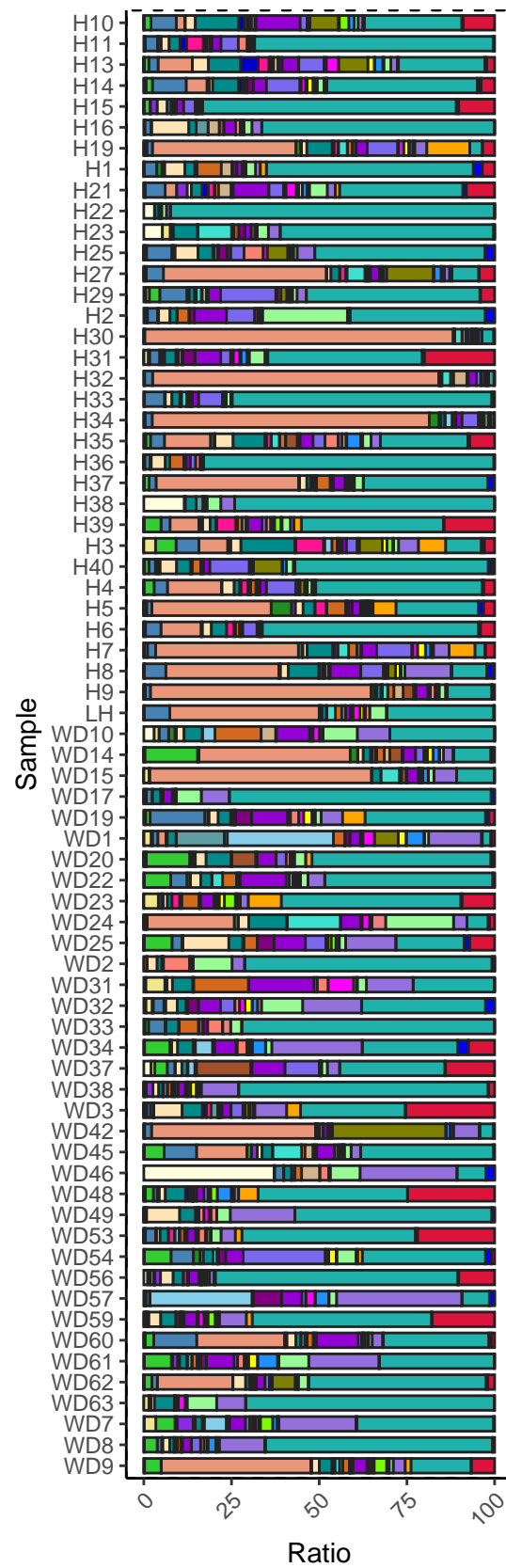

Supplement: Supplementary file 2 [file Data_Sheet_2.PDF]

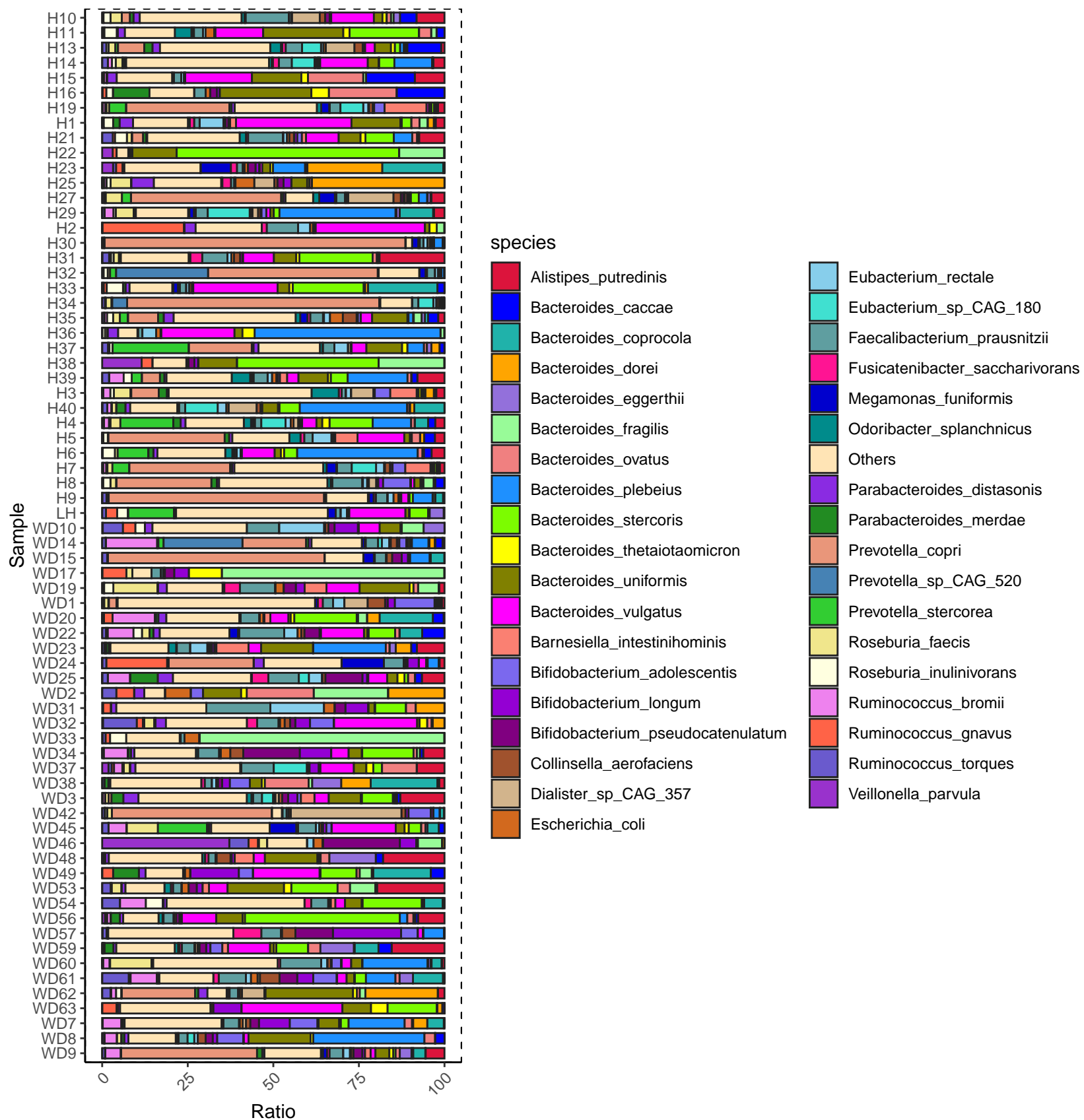

Supplement: Supplementary file 3 [file Data_Sheet_3.PDF]

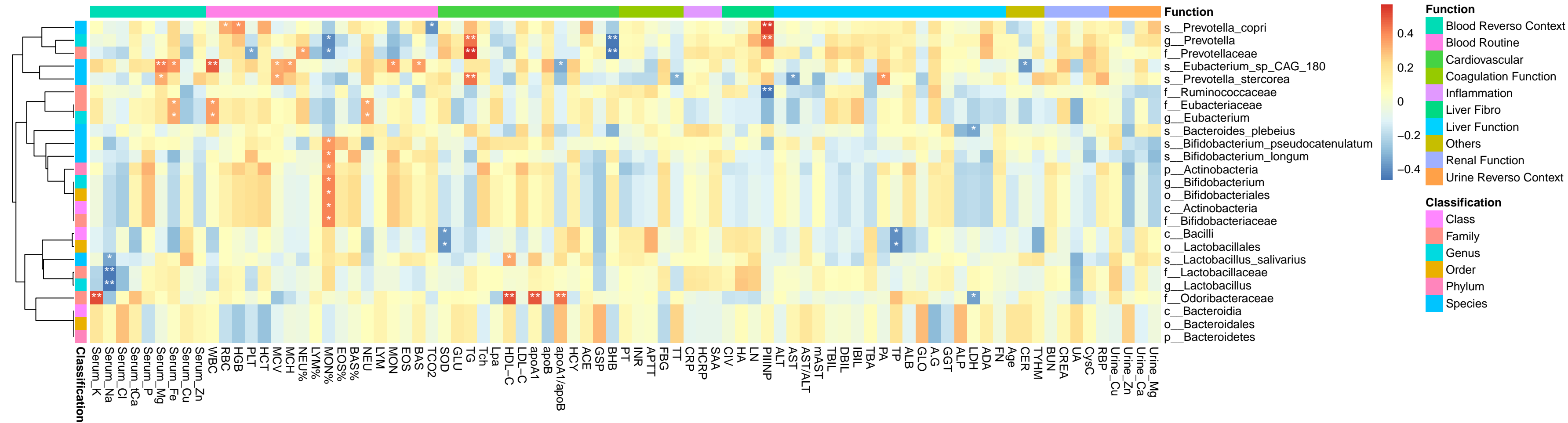

Supplement: Supplementary file 4 [file Data_Sheet_4.PDF]

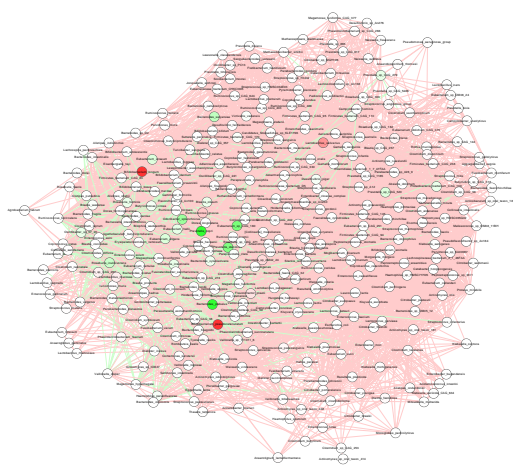

Supplement: Supplementary file 5 [file Data_Sheet_5.PDF]

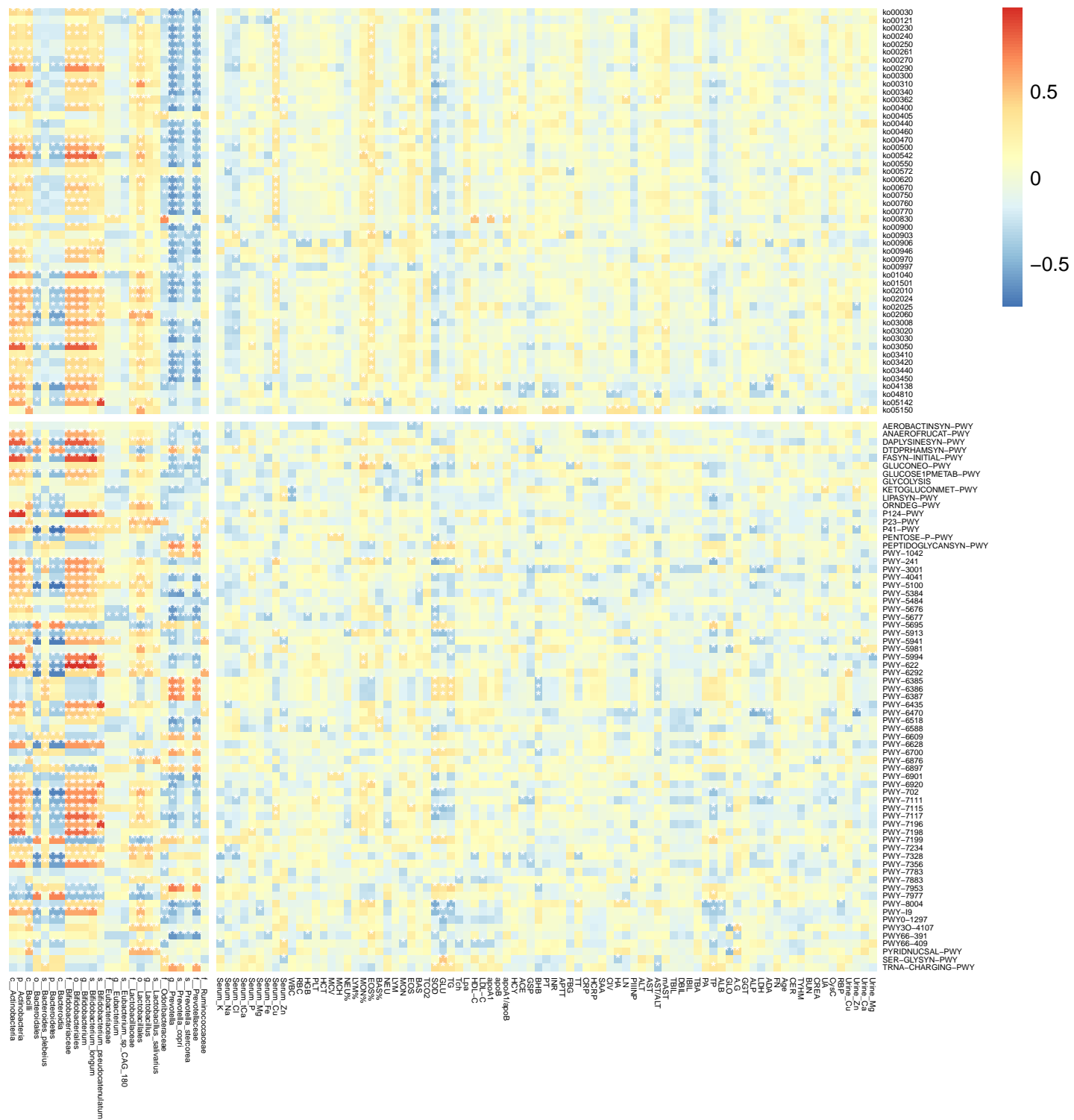

Supplement: Supplementary file 6 [file Data_Sheet_6.PDF]

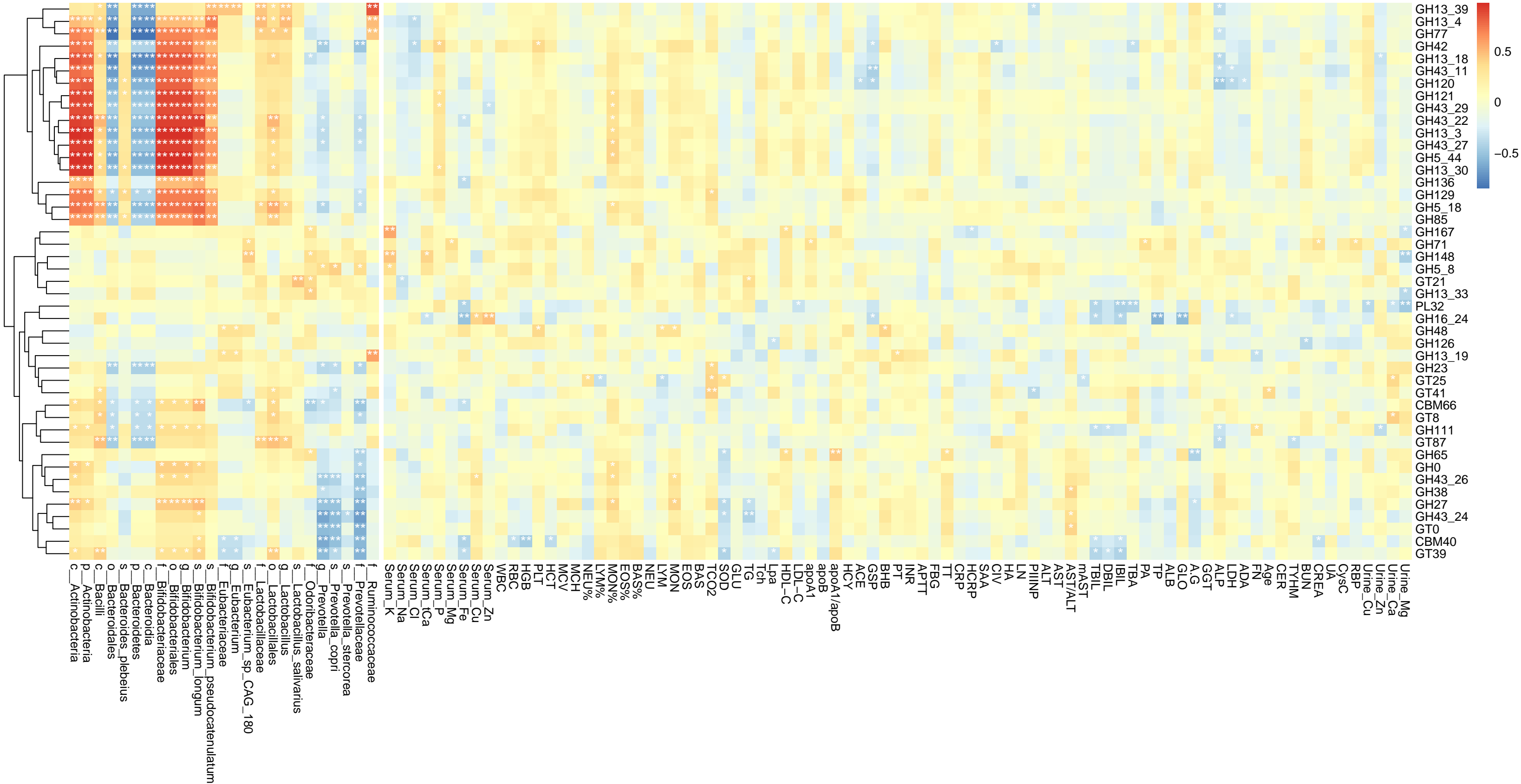

Supplement: Supplementary file 7 [file Data_Sheet_7.PDF]

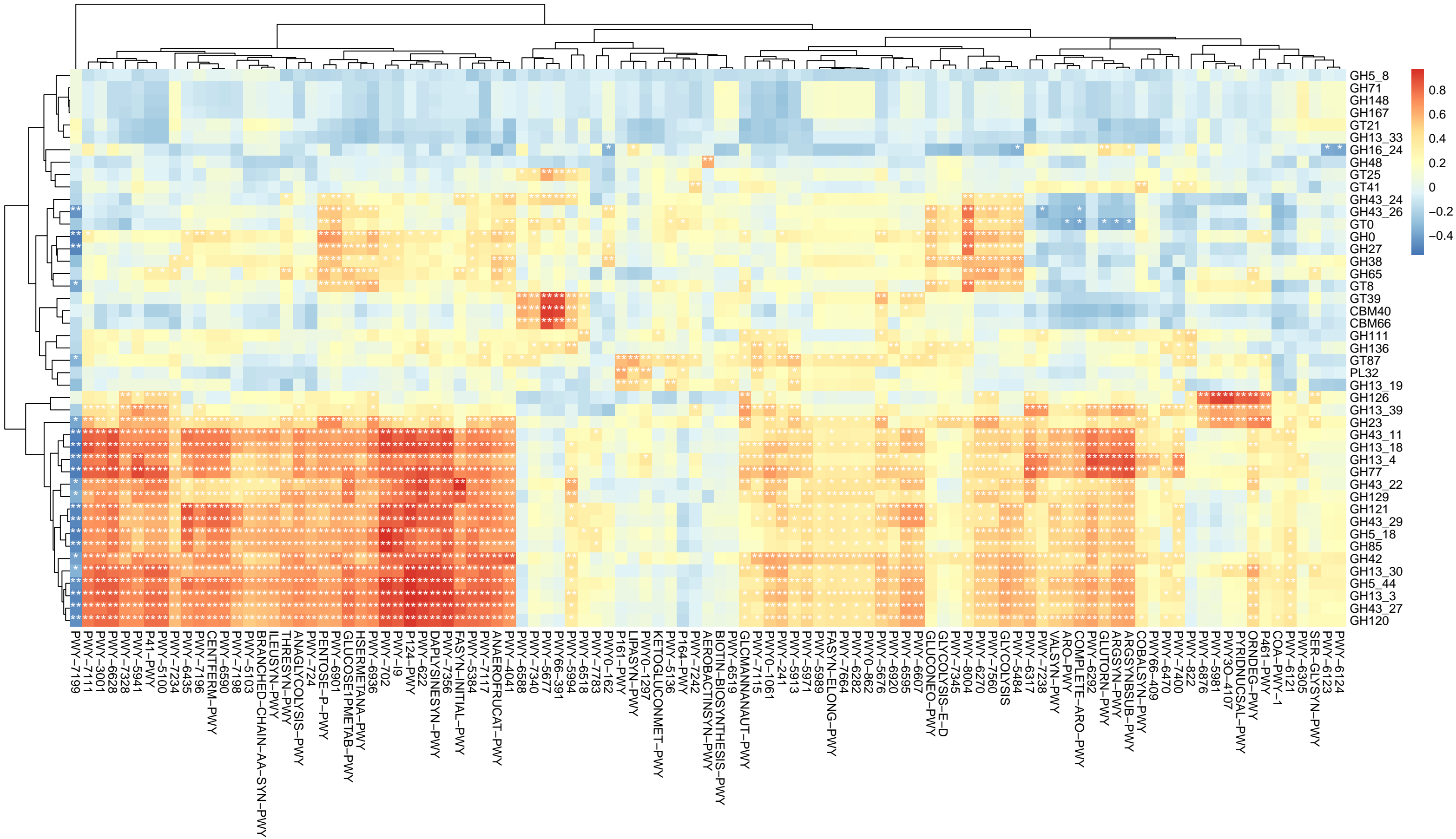

Supplement: Supplementary file 8 [file Data_Sheet_8.PDF]

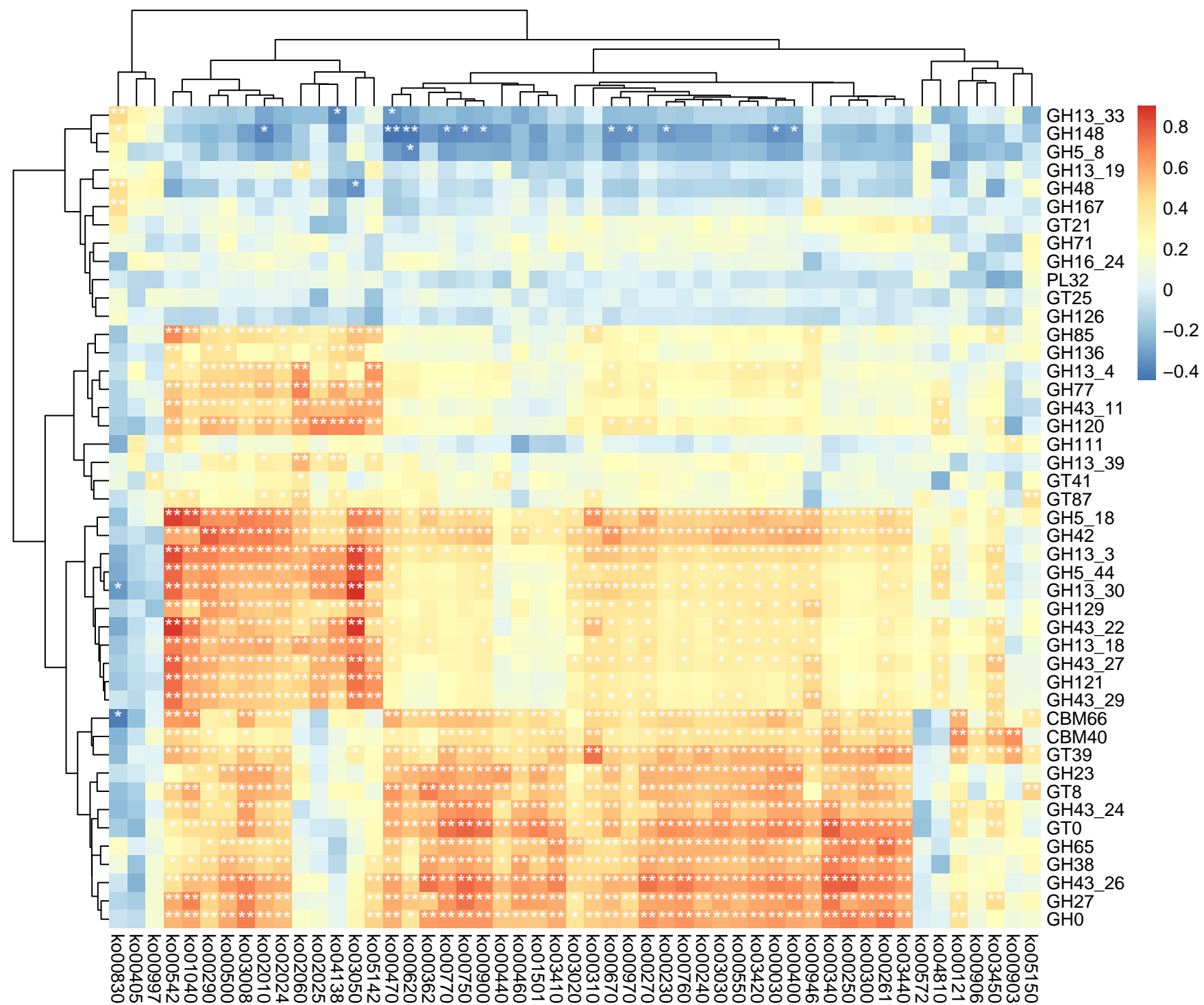

Supplement: Supplementary file 9 [file Data_Sheet_9.PDF]

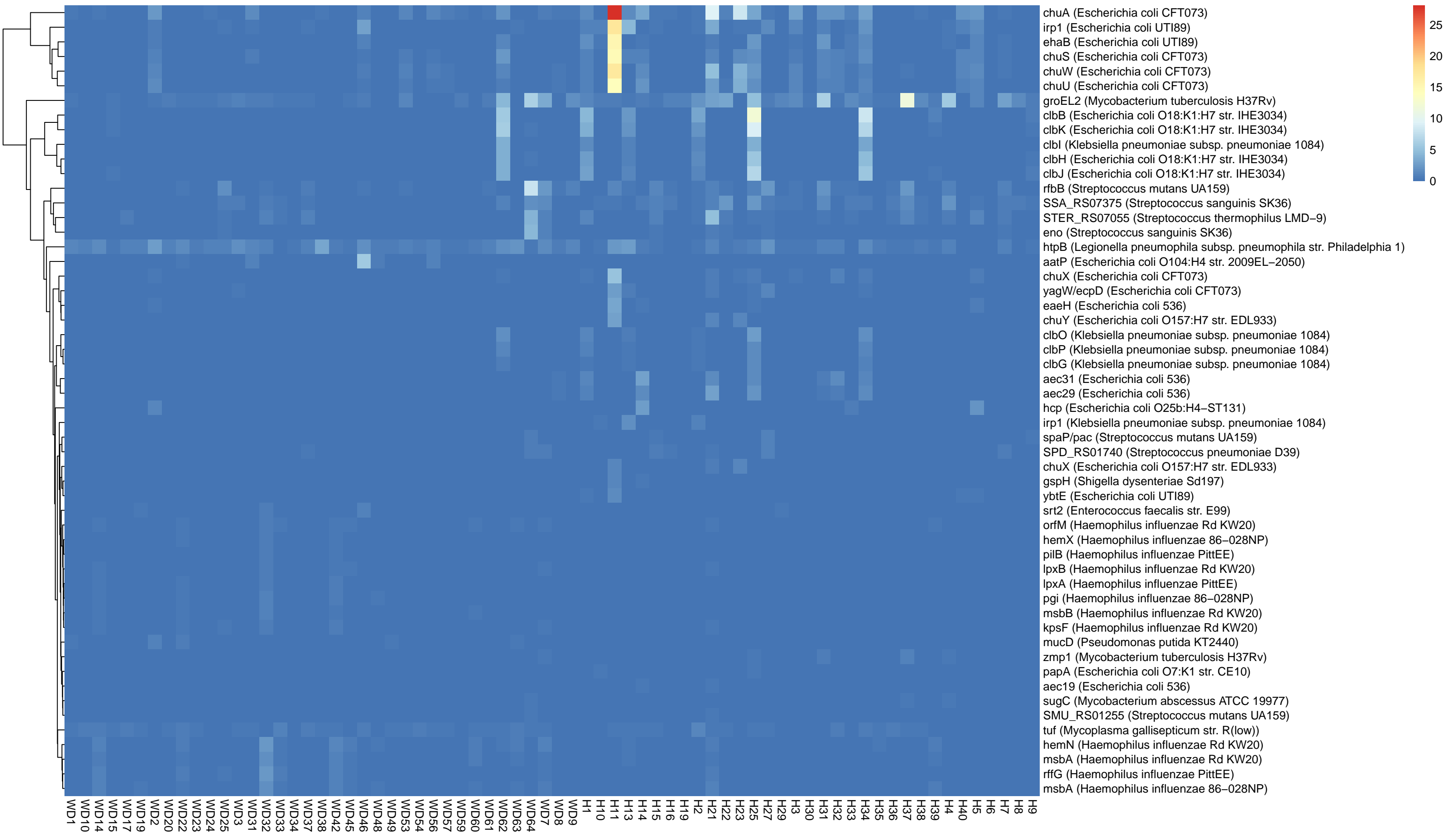

Supplement: Supplementary file 10 [file Data_Sheet_10.PDF]

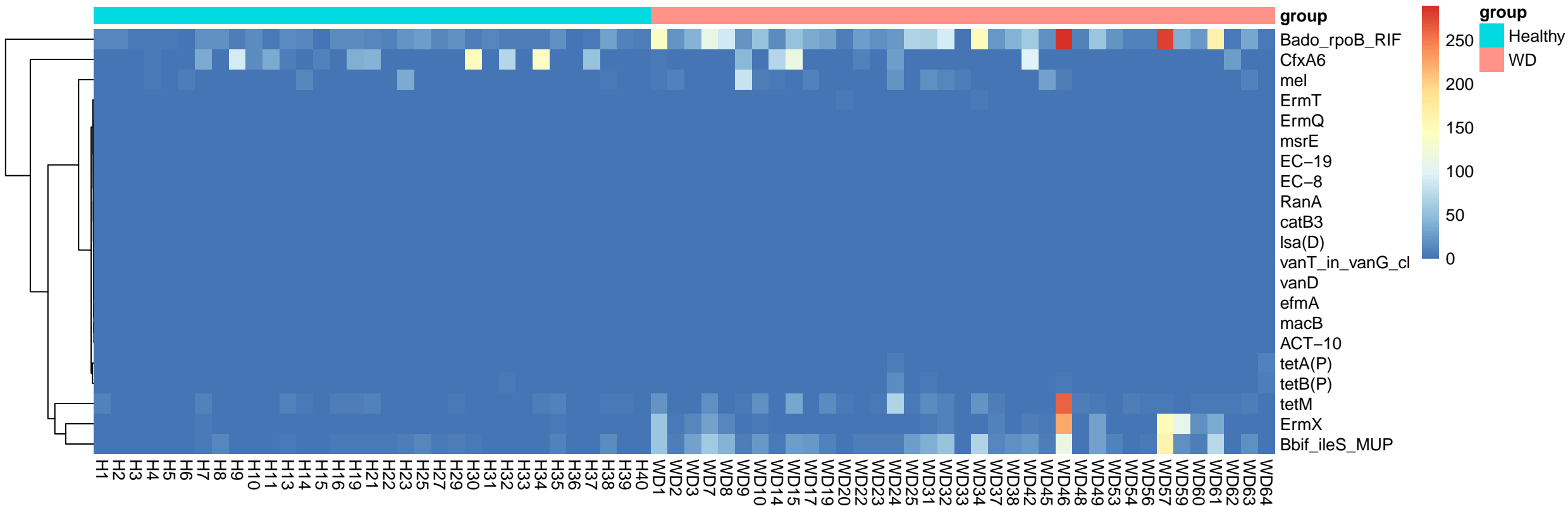

Supplement: Supplementary file 11 [file Data_Sheet_11.PDF]
